# Engineering Photonic Pigments From Titania–Block Copolymer Composites

**DOI:** 10.1002/marc.70359

**Published:** 2026-07-14

**Authors:** Niklas Rocca Schwarz, Jorge Larios, Antoine Scalabre, Matthias Saba, Ullrich Steiner, Andrea Dodero

**Affiliations:** ^1^ Adolphe Merkle Institute University of Fribourg Fribourg Switzerland; ^2^ NCCR Bio‐inspired Materials University of Fribourg Fribourg Switzerland; ^3^ Department of Chemistry University of Fribourg Fribourg Switzerland

**Keywords:** block copolymers, non‐iridescent, photonic pigments, structural color, titania nanoparticles

## Abstract

Structural color pigments based on block copolymer self‐assembly offer non‐fading color. However, their brightness and saturation are often limited by a low‐refractive index contrast and incoherent scattering arising from disorder. Here, we engineer non‐iridescent photonic pigments by incorporating ligand‐functionalized TiO_2_ nanoparticles into the poly(2‐vinylpyridine) domains of onion‐like polystyrene‐*block*‐poly(2‐vinylpyridine) microspheres fabricated by emulsion‐confined self‐assembly. Thermally assisted solvent evaporation improves long‐range lamellar order at high nanoparticle loadings, suppressing disorder and enabling strong Bragg reflections in individual microspheres. Quantitative loading analysis reveals high incorporation efficiency up to large selective TiO_2_ fractions. Ultra‐small angle X‐ray scattering and electron microscopy demonstrate that increasing the nanoparticle content reduces lamellar periodicity and couples refractive index enhancement to a blue‐shift. By combining selective swelling with molecular‐weight tuning, we shift the Bragg peak across the visible spectrum and fabricate pigments with enhanced saturation and luminance compared to nanoparticle‐free references. We use optical modeling with a transfer‐matrix method and Ewald‐sphere analysis to interpret the measurement geometry, guide the design process, and establish links between the objective numerical aperture, mode collection, and perceived coloration. These results provide practical design rules for high‐performance structural pigments by coupling control over ordering, scattering, and composite refractive index contrast.

## Graphical abstract

Ligand‐functionalized TiO_2_ nanoparticles selectively partition into P2VP‐rich lamellae of onion‐like PS‐*b*‐P2VP microspheres, increasing refractive index contrast while preserving concentric order through controlled self‐assembly. The resulting dry photonic pigments display defined Bragg reflections and angle‐robust structural color. Tunable molecular weight and selective swelling adjust lamellar periodicity across the visible range, linking composition, ordering, and optical response.

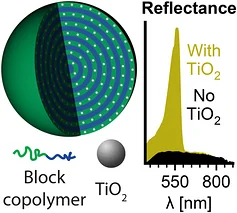

## 1 Introduction

Structural color arises from the interaction of light with periodic dielectric architectures commonly known as photonic crystals. These structures have lattice parameters within the visible‐light range (i.e., hundreds of nanometers) [1]. Because color is encoded in the structure rather than the chemistry, this class of materials is a promising source of vivid, non‐fading coloration that rivals traditional absorbing pigments and dyes in saturation and brightness [2]. However, translating photonic‐crystal concepts into practical structural pigments remains challenging. Fabrication methods must be scalable, colors must be bright and saturated under diffuse illumination, and, critically for many applications, the appearance must be largely non‐iridescent (i.e., colors must be independent of the viewing angle). Top‐down approaches, such as photolithography, are inherently slow and costly. Moreover, scaling them up for three‐dimensional photonic crystal fabrication is particularly challenging [3]. Conversely, bottom‐up self‐assembly enables inexpensive fabrication of intricate periodic structures that are currently used in photonic nanodevices [4, 5], solar‐cell technologies [6], and optical metamaterials [7]. In this context, block copolymers (BCPs) are a promising platform because they can spontaneously self‐assemble into periodic nanostructures whose characteristic length scales match visible wavelengths [8, 9], therefore being inherently suitable for photonic materials.

BCPs consist of two or more chemically distinct polymer blocks that are covalently linked within a single chain. While the blocks are thermodynamically driven to demix, they cannot macroscopically phase‐separate due to their tethering. Instead, BCPs undergo microphase separation into ordered, periodic nanodomains. The resulting equilibrium morphologies (e.g., lamellae, cylinders, and spheres) are primarily determined by the block volume fractions and segregation strength, while the characteristic period is primarily controlled by chain length and can be further adjusted by processing conditions such as solvent selectivity, temperature, and annealing history [10]. These factors provide a direct and practical means of controlling both symmetry and length scale, ensuring strong interaction with visible light [11]. Additionally, selective swelling or additive partitioning can be used to adjust the domain periodicity during processing without redesigning the starting polymer. BCPs also retain the practical advantages of polymers, including compatibility with solution processing and scalable fabrication. These properties make block copolymers a versatile bottom‐up platform for photonic materials and visible structural color [4, 9].

Crucially, BCP self‐assembly is highly sensitive to boundary conditions, and confinement provides an additional means for directing structure formation beyond what is typically available in bulk materials [12]. As a matter of fact, when assembly occurs in finite volumes or near interfaces, surface energy and geometry can influence domain orientation, thereby altering defect formation and annealing pathways. These changes can stabilize curved or radially organized morphologies [13]. For example, in evaporating emulsion droplets, the soft interface of the droplet determines the orientation of lamellae‐forming BCPs. In particular, the existence of a selective interface drives the formation of concentrically layered (onion‐like) microspheres with a periodically stratified refractive index profile [14]. This confinement‐directed architecture is particularly desirable for pigment applications because it creates a more angle‐robust appearance under broad illumination. Indeed, unlike planar multilayers, which have dominant Bragg reflections, a stratified sphere has many local layer orientations that interact with incident and reflected light. These interactions can suppress strong iridescence and produce pigment‐like structural color [15].

While previous studies focused on understanding the formation mechanism of such unique structures, our work aims to establish design rules for non‐iridescent photonic pigments that maximize saturation and brightness. Building on the droplet‐confined self‐assembly approach first introduced by Yang et al. [16], we fabricated polystyrene‐*b*‐poly(2‐vinylpyridine) (PS‐*b*‐P2VP) microspheres with an onion‐like structure. We achieved this by emulsifying a BCP chloroform solution with an aqueous poly(vinyl alcohol) (PVA) surfactant phase. The color response of these BCP spheres can be approximated by Bragg's law [17]. Thus, as the optical path length increases, the wavelength reflection shifts toward the red due to either a larger refractive index or thicker lamellae. In practice, efficiently accessing the full visible color range requires controlling both contributions. Yang et al. demonstrated structural color tuning via the selective swelling of P2VP layers in ethanol/water mixtures, which links periodicity changes directly to the optical response [16]. Miyake et al. showed that the Bragg peak can be red‐shifted by selecting a higher degree of polymerization, corresponding to longer polymer chains, which yields extended domain spacing L [18]. Alternatively, periodicity can be modified by exploiting “swelling additives” that selectively increase the volume of one microphase in the BCP self‐assembled morphology. Yang et al. demonstrated that adding polystyrene (*h*PS) homopolymer and 3‐n‐pentadecylphenol (PDP) to PS‐*b*‐P2VP onion‐like particles increases the thickness of PS and P2VP layers, respectively, due to their selective segregation. This leads to a redshift of the observed color [19]. Notably, the pronounced structural color response of pristine PS‐*b*‐P2VP photonic pigments is typically observed in the wet state, whereas dry particles often exhibit substantially weaker color.

These elegant works laid the foundations for structurally colored pigments. However, a central limitation of pristine PS‐*b*‐P2VP and other common BCPs is their low refractive index contrast (e.g., $n_{P2\mathrm{VP}}/n_{\mathrm{PS}}=1.02$ at 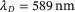 [20]), which limits reflectance intensity and color lightness (relative luminance in the CIELAB color space [21]). Increasing the refractive index contrast between alternating layers is a direct route to brighter structural colors. Selectively incorporating high‐index nanoparticles, such as titania or zirconia, into a single BCP microdomain enhances the optical performance of distributed Bragg reflectors [22]. However, nanoparticle loading is not merely an optical modification. Nanoparticles can strongly influence BCP thermodynamics and mechanics, affecting domain spacing, degree of order, defect density, and swelling behavior. These structural changes can shift hue and modify saturation and lightness. Thus, refractive index engineering and morphology control are intrinsically intertwined. Previous research on nanoparticle‐BCP composites has shown that nanoparticle distribution depends on particle size and interactions with the polymer chains [23, 24]. For example, Warren et al. found that the distribution of silica nanoparticles in poly(isoprene‐*block*‐ethyleneoxide) (PI‐*b*‐PEO) depends on the ratio of nanoparticle size to domain dimension. The nanoparticles can be distributed within a single block, segregated between the core and the shells, or concentrated in the core of onion‐like structures [25].

Although substantial research has examined the influence of nanoparticles on morphology, domain spacing, and nanoparticle partitioning of BCP composites, few studies have investigated these effects in BCP‐based photonic structures with high nanoparticle loading. The optical performance of these structures depends on the refractive index contrast, structural periodicity, and disorder‐induced scattering. Simultaneously engineering the scattering properties, structural order, and environmental responsiveness of onion‐like photonic pigments requires a combined experimental and computational approach.

Here, we investigated TiO_2_‐PS‐*b*‐P2VP photonic pigments as a model system to reveal the coupled optical and structural consequences of nanoparticle incorporation into onion‐like self‐assembled BCP morphologies. We selected TiO_2_ nanoparticles because they have a high refractive index in the visible spectrum (n(589nm)=2.51 [26]), negligible optical extinction, and are compatible with polymer processing when appropriately surface‐functionalized. The primary goal of TiO_2_ incorporation was to enhance the dielectric contrast between the alternating lamellae by selectively raising the refractive index of the P2VP‐rich domains. This improves the intensity and quality of the photonic response. We also examined how this refractive‐index engineering strategy affects lamellar spacing, ordering, and swelling.

Our fabrication process follows the droplet‐confined co‐self‐assembly route previously described by Bertucci et al. [27]. As shown in Figure 1A, an organic phase containing BCPs and nanoparticles is emulsified in water. Solvent evaporation and preferential interfacial interactions [28, 29, 30, 31] lead to the self‐assembly of onion‐like microspheres (Figure 1B). These interactions are crucial for obtaining periodic shells rather than parallel lamellae of alternating polymer microdomains [32]. Using PS‐*b*‐P2VP spheres as a lamellar scaffold enables us to achieve high‐selective TiO_2_ loadings targeted to the P2VP domains. We quantify how nanoparticle fraction correlates with lamellar shrinkage and order‐disorder transitions, and we correlate these morphological changes with reflectance spectra and visual appearance to identify processing and composition regimes that enhance or degrade color quality.

**FIGURE 1:**
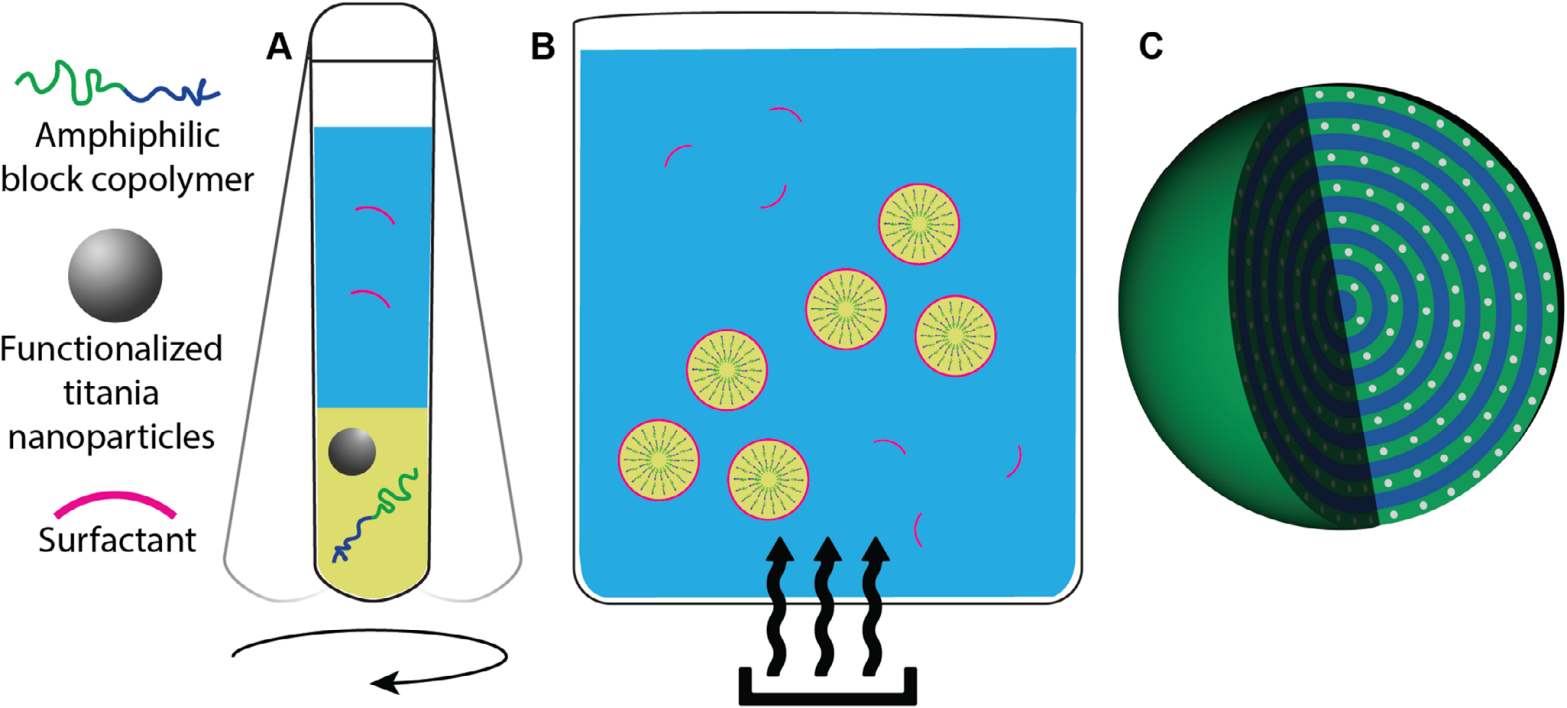
Photonic Pigment Fabrication Protocol. BCPs self‐assemble confined within emulsion droplets to form concentric lamellar microparticles. (A) illustrates the vortex‐assisted emulsification of functionalized titania nanoparticles and BCPs dispersed in an organic phase within an aqueous surfactant phase. (B) illustrates how increasing the temperature enhances the thermodynamics of self‐assembly as the organic phase evaporates. (C) shows the final, ideal structure with defect‐free, alternating shells and titania nanoparticles dispersed and localized within the high‐refractive index polymer microdomains.

Additionally, we perform transfer‐matrix calculations to interpret spectral measurements. Planar transfer‐matrix calculations provide a practical, first‐order estimate of peak position by approximating concentric lamellae as locally planar layers. Reciprocal‐space Ewald sphere considerations explain how the collection numerical aperture affects the measured spectra.

Overall, our results establish links between the distribution of titania nanoparticles, structure formation, and the resulting scattering that gives rise to the perceived color. These findings contribute to the development of non‐iridescent structural pigments. The selectively incorporated titania volume fractions achieved here are, to our knowledge, among the highest values reported for self‐assembled block copolymer systems. The high nanoparticle loadings significantly improve the color saturation and brightness of BCP microspheres.

## 2 Results and Discussion

### 2.1 Selective Titania Incorporation in PS‐*b*‐P2VP

The goal was to increase reflectance intensity and consequently color lightness by incorporating TiO_2_ nanoparticles into the high‐index polymer microdomains within onion‐like BCP microspheres. High loading and long‐range lamellar order require stable nanoparticle dispersion to prevent agglomeration and strong, preferential interactions with one polymer block [33, 34]. Therefore, we first screened particle surface chemistries using gallic acid‐functionalized TiO_2_ nanoparticles (i.e., hydrophilic shell) and commercially available TiO_2_ nanoparticles with hexylamine and dodecylphosphonic acid ligands (TiO_2_‐HA/DA) (i.e., hydrophobic shell). We investigated the combination of titania nanoparticles with three commercial linear diblock copolymers: PS‐*b*‐P2VP, P2VP‐*b*‐PMMA, and PS‐*b*‐PMMA. Pristine BCP onion‐like particles are shown as a reference in Figure S1A–C, respectively. Polystyrene and poly(2‐vinylpyridine) have similar refractive indices of 1.59 and 1.62 at a wavelength of 589 nm [20], respectively, while poly(methyl methacrylate) has a lower refractive index of 1.49 [35]. Therefore, BCPs containing PMMA are, in principle, more attractive due to their relatively high refractive index contrast with PS and P2VP. However, a minor drawback of PS‐*b*‐PMMA is that the low‐refractive index PMMA is expected to form the outer shell of the self‐assembled microspheres. This reduces the Bragg peak intensity, particularly for structures comprising only a limited number of layers. This behavior is consistent with the thin‐film interference theory. Because distributed Bragg reflector layers act as optical‐admittance inverters, the effective input admittance (and thus the impedance mismatch to air) depends strongly on the terminating layer. Consequently, a low‐index termination (PMMA) produces a smaller mismatch than a high‐index termination, thereby lowering the Bragg‐peak reflectance [36].

First, gallic acid‐functionalized titania nanoparticles were selectively loaded into alternating concentric lamellae of PS‐*b*‐PMMA photonic microspheres (Figure S2A). However, significant agglomeration of the nanoparticles occurred when the same protocol was applied to PS‐*b*‐P2VP (Figure S2B) or P2VP‐*b*‐PMMA (Figure S2C), as observed in the reported FIB‐SEM cross‐sections. We hypothesize that gallic acid‐functionalized TiO_2_ forms stronger, more specific bonds with the pyridyl groups of P2VP. The pyridine nitrogen can participate in directional hydrogen bonding. Under sufficiently acidic local conditions, partial protonation can further strengthen association through acid‐base interactions [37]. Such strong adsorption may promote interparticle attractions, overcome steric stabilization, and favor flocculation [38]. PMMA primarily provides ester carbonyl acceptors and lacks a comparably basic site. Therefore, its interactions with gallic‐acid groups are expected to be weaker and less associative, reducing the tendency toward adsorption‐driven aggregation [39].

The use of gallic acid‐functionalized TiO_2_ nanoparticles with polystyrene‐*b*‐poly(methyl methacrylate) was not thoroughly investigated for two reasons. First, the resulting small lamellar spacing (∼98nm) produced a Bragg peak in the UV spectral range, estimated at around 314 nm. While increasing the molecular weight of the polystyrene‐*b*‐poly(methyl methacrylate) block copolymer beyond 235‐*b*‐263 kDa would shift the Bragg peak into the visible range, no commercially available materials meet this criterion. Second, the spatial distribution of nanoparticles within the block copolymer domains remains poorly understood and is not yet fully controllable. For polystyrene‐*b*‐poly(methyl methacrylate), the nanoparticles should localize within the PS lamellae to increase the refractive index contrast between the alternating layers. However, the nanoparticles also partition into the PMMA lamellae, which diminishes the effective optical contrast and constrains the strength and purity of the reflected color.

Consequently, commercial TiO_2_‐HA/DA nanoparticles were primarily investigated. We achieved domain‐selective loading of $f_{\mathrm{NP}}=20\;\mathrm{wt}.\%$ in PS‐*b*‐P2VP and P2VP‐*b*‐PMMA microspheres without agglomeration. FIB‐SEM cross‐section of self‐assembled microspheres confirmed this finding and revealed their concentric lamellar internal structure (Figure S3A,B, respectively). Conversely, agglomeration of TiO_2_‐HA/DA nanoparticles was evident in polystyrene‐*b*‐poly(methyl methacrylate) microspheres (Figure S3C). The compatibility of TiO_2_‐HA/DA with PS‐*b*‐P2VP and P2VP‐*b*‐PMMA, but not with PS‐*b*‐PMMA, suggests that these nanoparticles have a higher affinity for P2VP‐containing domains than for PS‐ or PMMA‐rich domains. We attribute this selectivity to favorable interactions between the TiO_2_‐HA/DA surface/ligand shell and the pyridyl groups of P2VP. These may include weak acid‐base, hydrogen‐bonding, coordination‐like, or dipolar interactions involving residual polar TiO_2_ surface sites, surface hydroxyl groups, or polar ligand headgroups. Such interactions reduce the interfacial penalty for incorporating the nanoparticles into the P2VP‐rich domains and stabilize them against aggregation during solvent removal and microphase separation. This interpretation is consistent with previous reports on P2VP‐containing block copolymer/nanoparticle composites, including systems containing hydrophobically capped inorganic nanocrystals [34] where the final nanoparticle localization is governed by the balance of surface/ligand‐block interactions.

Poly(2‐vinylpyridine)‐*b*‐poly(methyl methacrylate) microspheres prepared from 235‐*b*‐220 kDa block copolymers exhibited a lamellar spacing of $L_{0}∼96\;\mathrm{nm}$, corresponding to a Bragg peak in the UV at 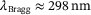. Therefore, as was observed for polystyrene‐*b*‐poly(methyl methacrylate), higher molecular weights are required to shift the peak into the visible range. However, no commercially available system exists. PS‐*b*‐P2VP was chosen despite its small initial refractive index contrast and poor ability to achieve high color lightness. Several high‐molecular‐weight PS‐*b*‐P2VP polymers are commercially available for producing a Bragg peak in the visible range, and incorporating nanoparticles into them exhibits no signs of agglomeration. Notably, for similar molecular weights, PS‐*b*‐P2VP leads to a much higher lamellar periodicity than polystyrene‐*b*‐poly(methyl methacrylate) (160 nm for 380 kDa vs. 88 nm for 498 kDa, as determined from USAXS scattering (Figure S4).

This difference likely stems from the fact that the Flory‐Huggins interaction parameter of P2VP‐*b*‐PMMA is approximately three times lower than that of PS‐*b*‐P2VP (see Section 4.1). This difference affects the domain spacing, as expressed by the strong‐segregation relation,

(1) $$L_{0}∼bN^{2/3}\chi_{\mathrm{eff}}^{1/6},$$

where b is the statistical segment length and N is the overall degree of polymerization [8]. A lower χ value indicates greater compatibility between the two polymer blocks, enhancing miscibility and weakening segregation. Consequently, interfacial tension decreases, and the lattice constant L is reduced.

#### 2.1.1 Processing and Thermal Annealing to Optimize Long‐Range Order

Increasing the loading of nanoparticles modifies the self‐assembly process. Exceeding a critical mass fraction can result in the formation of disordered lamellae instead of concentric ones. A significant refractive index contrast enhances reflectance and, consequently, color lightness. Therefore, highly selective NP incorporation is only desirable if lamellar order is preserved. Figure 2A illustrates the formation of a partially disordered structure during room‐temperature assembly at a $f_{\mathrm{NP}}$ value of 25 %. This is equivalent to a selective nanoparticle volume fraction of $\phi_{\mathrm{NP}}=19.4\;\%$ in P2VP. Figure S5 shows FIB‐SEM cross sections of PS_185kDa_‐*b*‐P2VP_195kDa_ microparticles with increasing nanoparticle content (A–C). The figure clearly shows the formation of disordered morphologies at high loadings. The mass fraction is defined as the ratio of the mass of the nanoparticles to the mass of the BCP. The volume fraction is defined as the volume of the nanoparticles divided by the combined volume of P2VP and nanoparticles,

(2) $$f_{\mathrm{NP}}=\frac{m_{\mathrm{NP}}}{m_{\mathrm{BCP}}},$$

(3) $$\phi_{\mathrm{NP}}=\frac{V_{\mathrm{NP}}}{V_{\mathrm{NP}}+V_{P2\mathrm{VP}}}.$$

With an identical sample composition, increasing the temperature at which the self‐assembly occurs to 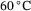 results in enhanced internal order, most likely due to modified assembly kinetics, as illustrated in Figure 2B. Additionally, to ensure slower evaporation, the organic solvent was exchanged from chloroform to 1,2‐dichloroethane (DCE). DCE has a higher boiling point (i.e., $83.5^{\circ }C$) than chloroform (i.e., $61.2^{\circ }C$), which slows evaporation under these conditions.

**FIGURE 2:**
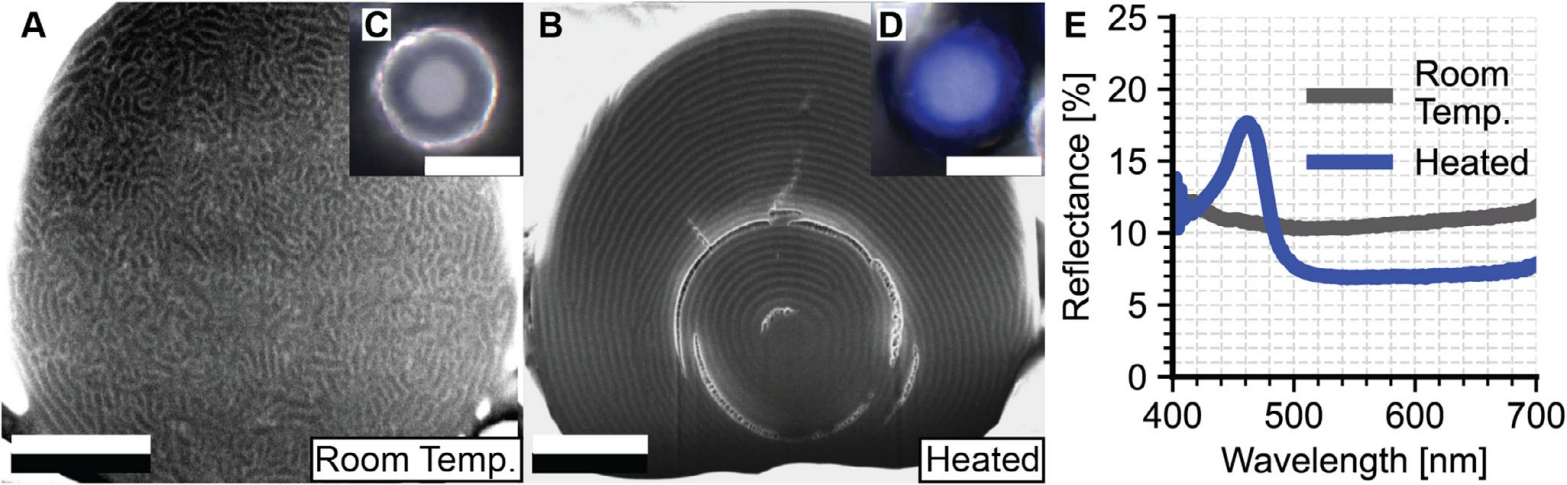
Effect of temperature on the self‐assembly process. (A) FIB‐SEM cross‐section of a microsphere prepared through self‐assembly at room temperature and (C) associated optical microscope micrograph. (B,D) show a microsphere prepared at 60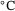. The scale bars are 2 μm in (A,B), and 20 μm in (C,D). (E) shows reflectance curves demonstrating increased lightness and color saturation for self‐assembly at high temperatures.

Although the system segregation strength χN must be sufficiently high to sustain BCP microphase separation, the formation of well‐ordered concentric lamellae is often limited by kinetics. During solvent removal and confinement within microspheres, the chains may become immobilized before defects and grain boundaries anneal. This results in imperfectly ordered textures. Furthermore, incorporating nanoparticles can exacerbate kinetic trapping by increasing the effective viscosity and pinning interfaces, thereby perturbing the mixture's segregation strength. Consequently, an elevated temperature is essential to restore sufficient mobility and recover highly ordered, concentric lamellae. Increasing the annealing temperature enhances segmental mobility, reduces viscosity, and enables defect annihilation, grain coarsening, and reorientation toward equilibrium onion‐like lamellar morphology [40, 41].

The enhancement in internal order is clearly reflected in the optical response of dried microparticles. Disorder induces incoherent scattering, resulting in a whitish appearance (Figure 2C). In contrast, high structural order significantly improves color saturation (Figure 2D). Figure 2E shows the spectral difference between incoherent scattering and the formation of a well‐defined Bragg peak, a characteristic of an ordered photonic crystal. Therefore, self‐assembly at elevated temperature is critical for improving structural order and optimizing color properties, such as saturation and lightness, at high nanoparticle loading.

TEM and FIB‐SEM cross‐sectional micrographs of samples prepared at 60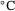 provide direct, real‐space evidence of improved ordering of the concentric lamellae compared to the corresponding room‐temperature samples (Figure S6 and Figure 2). The elevated‐temperature samples, in particular, exhibit more continuous lamellar domains and reduced disorder, consistent with enhanced chain mobility during droplet self‐assembly. The corresponding optical response, characterized by a more defined Bragg reflection and improved color saturation, further supports this interpretation. Based on these observations and the correlated optical response, USAXS measurements on the elevated‐temperature samples are not expected to alter the conclusion that a higher self‐assembly temperature improves lamellar ordering at high nanoparticle loading.

#### 2.1.2 Effect of Titania Incorporation on Self‐Assembly and Domain Periodicity

Interestingly, lamellar periodicity *decreases* with the addition of increasing amounts of TiO_2_‐HA/DA nanoparticles. Figure 3A shows ultra‐small angle X‐ray scattering (USAXS) measurements of PS_185kDa_‐*b*‐P2VP_195kDa_ microspheres with titania volume fractions in the range 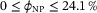. As the nanoparticle content increases, the scattering peak shifts toward higher scattering vectors q, indicating a reduction in lamellar thickness. The thickness decreases from approximately 159 nm in the pristine state to 144 nm at $\phi_{\mathrm{NP}}=5.0\;\%$ and decreases further to 90 nm at $\phi_{\mathrm{NP}}=24.1\;\%$. Additionally, a progressive peak broadening correlates with increasing disorder, particularly beyond $\phi_{\mathrm{NP}}=15.0\;\%$. Please note that the USAXS measurements were performed on samples prepared at room temperature.

**FIGURE 3:**
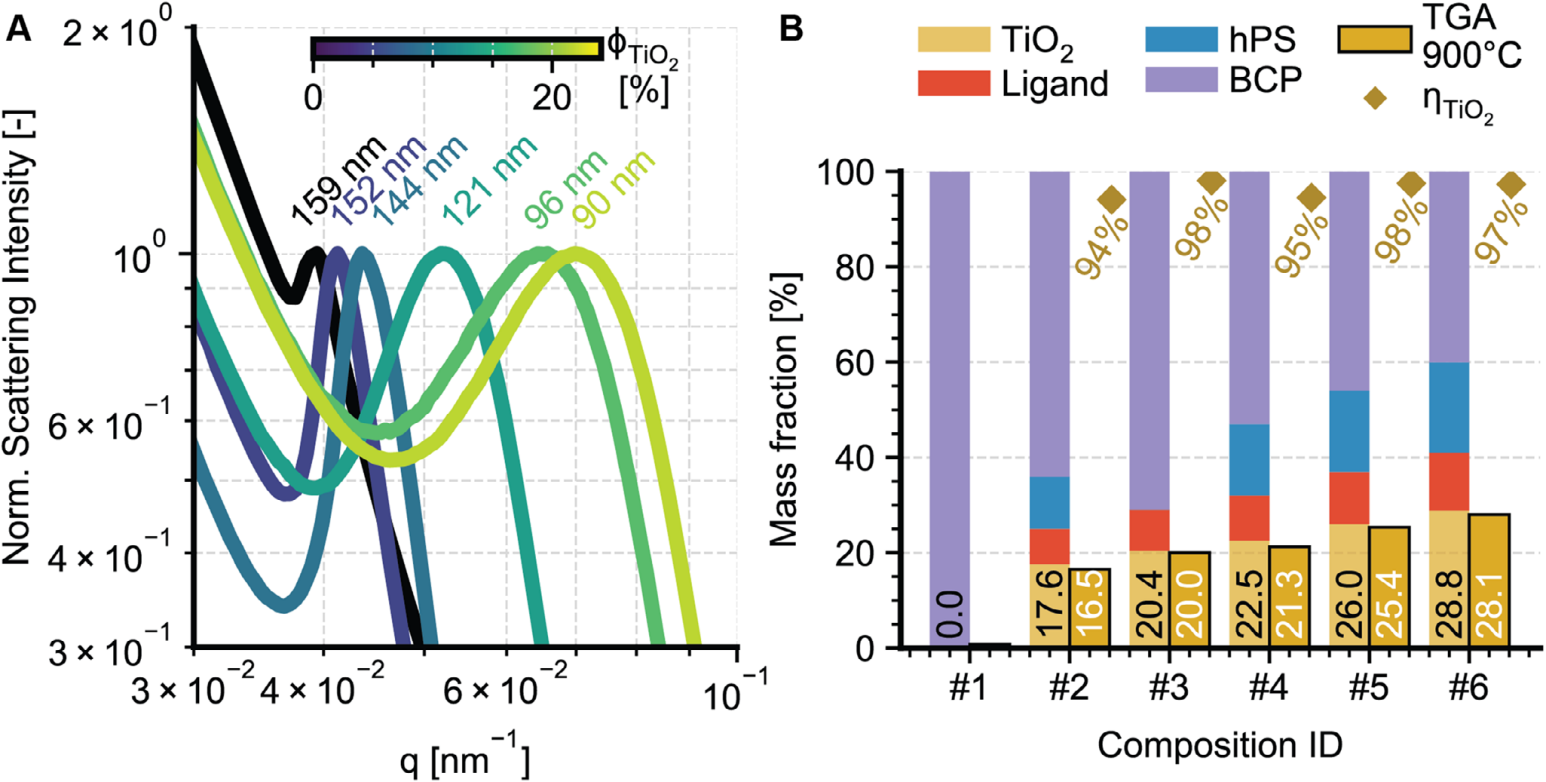
TiO_2_ is efficiently incorporated into PS‐*b*‐P2VP microspheres, significantly affecting the spacing of lamellar domains. (A) shows USAXS profiles of PS‐*b*‐P2VP microspheres with increasing titania content. The volume fraction of TiO_2_‐HA/DA within the P2VP microdomains is represented by $\phi_{TiO_{2}}$. As the loading of TiO_2_ nanoparticles increases, the lamellar domain size decreases accordingly, shifting from around 160 nm for pristine BCP to 90 nm at $\phi_{\mathrm{NP}}\;=\;24.1\;\%$. (B) shows thermogravimetric analysis profiles used to quantify effective TiO_2_ content for different microsphere compositions. The “TiO_2_ loading efficiency” $\eta_{{\mathrm{TiO}}_{2}}$ is defined in Equation 4. The term “ligand” refers to the initial mass minus the residual mass of hexylamine and dodecylphosphonic acid associated with the TiO_2_‐HA/DA nanoparticles upon heating to 600°C, as determined via TGA.

Transmission electron microscopy (TEM) cross‐section images (Figure S6) confirm the trend of lamellar shrinkage at high titania loadings, even under high‐temperature self‐assembly conditions. Figure S7 compares the change in lamellae thickness with respect to the titania volume fraction for USAXS vs. TEM measurements. The larger lattice constants determined from TEM may reflect the elevated‐temperature self‐assembly conditions used for these samples, which promote chain mobility and partial relaxation toward a more equilibrated lamellar spacing.

Contrary to the initial expectation that titania incorporation would thicken the lamellae, USAXS and TEM data clearly demonstrate the opposite. We attribute this behavior to the effect of the TiO_2_‐HA/DA nanoparticles on the effective thermodynamics and chain packing of the PS‐*b*‐P2VP system. Specifically, TiO_2_‐HA/DA nanoparticles preferentially interact with the P2VP‐rich domains. This interaction is expected to reduce the effective segregation strength between the PS and P2VP microphases. This reduction in segregation strength decreases chain stretching and leads to a smaller lamellar repeat distance. Jang et al. reported a similar observation in a different nanoparticle‐BCP system [42]. Furthermore, because TiO_2_ nanoparticles preferentially segregate within P2VP‐rich domains, pyridyl segments located near the nanoparticle surface experience restricted configurational freedom. This reduces the effective extension of the P2VP chains and promotes denser packing of the nanoparticle‐loaded domain during solvent removal. Therefore, we interpret the observed decrease in domain spacing as resulting from TiO_2_‐induced changes in the thermodynamics of the block copolymer and in the P2VP chain conformation/packing, rather than from a simple volume‐filling effect. It should be noted that the lamellar spacing in onion‐like microspheres is not expected to be perfectly uniform across the full particle radius. Even in pristine PS‐*b*‐P2VP microspheres, the inner lamellae can appear slightly thicker than the outer shells, reflecting the intrinsic radial inhomogeneity associated with spherical confinement and solvent removal. Therefore, the periodicities reported here should be interpreted as dominant/effective repeat distances. Nevertheless, TEM and FIB‐SEM cross‐sections do not indicate that the TiO_2_‐induced shrinkage is restricted to a specific radial region. Moreover, since the optical response of large microspheres is mainly governed by the outer ordered lamellae, possible moderate variations in the particle core do not affect the interpretation of the color response.

#### 2.1.3 Loading Efficiency

To evaluate the fraction of TiO_2_‐HA/DA nanoparticles incorporated into PS‐*b*‐P2VP microspheres after self‐assembly, thermogravimetric analysis (TGA) was performed on dry microspheres of varying compositions. Figure 3B shows the nominal mass composition of six samples and the residual mass after heating to 900°C. The residual mass corresponds exclusively to titania because it is the only component that does not combust or evaporate during thermal treatment. A loading efficiency parameter is defined as

(4) $$\eta_{{\mathrm{TiO}}_{2}}=\frac{m_{\mathrm{residual}}}{m_{{\mathrm{TiO}}_{2}}},$$

where the numerator represents the residual mass after heating the samples to 900°C. The denominator is the calculated nominal mass of titania based on the TiO_2_‐HA/DA nanoparticles added to the BCP solution prior to emulsification. The loading efficiency $\eta_{{\mathrm{TiO}}_{2}}$ remains consistently high across all compositions, with an average value exceeding 95 %. Thus, it can be assumed that titania incorporation remains unsaturated within the studied concentration range and no significant macrophase separation occurs.

#### 2.1.4 Nanoparticle Distribution and Refractive Index Enhancement

TEM cross‐sections of PS_185kDa_‐*b*‐P2VP_195kDa_ microspheres with increasing TiO_2_‐HA/DA loading provide sufficient contrast and spatial resolution to directly assess the NP distribution (Figure 4A–F). The NPs are not randomly dispersed throughout all samples but are instead localized in a periodic manner, primarily residing in every second lamellar domain. This alternating, domain‐selective localization is consistent with the preferential partitioning of TiO_2_‐HA/DA into only one of the two microphase‐separated blocks, as already discussed above.

**FIGURE 4:**
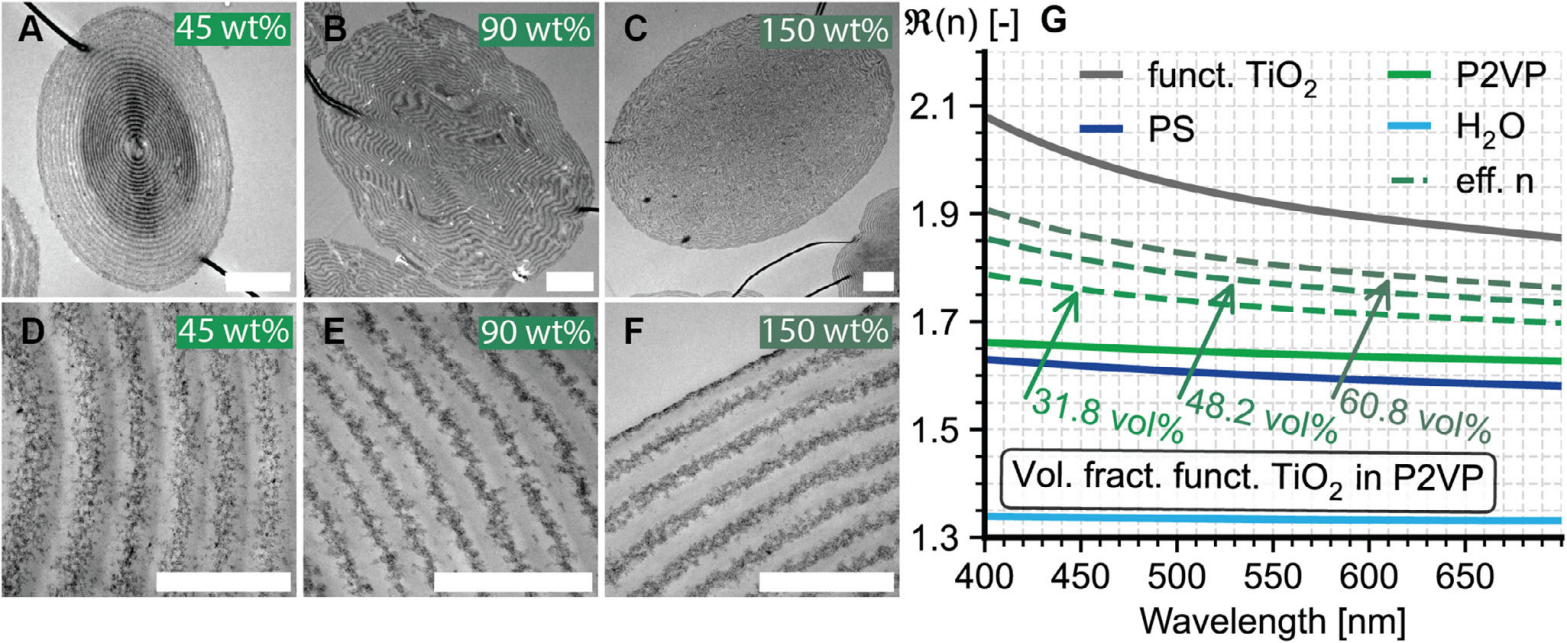
TEM micrographs (A–F) show cross‐sections of photonic microspheres at different loading fractions. They are shown as the mass ratio $f_{\mathrm{NP}}$ of the added nanoparticles to BCP, as defined in Equation 2 (Table S1 relates mass ratio and volume fractions for selected values). TEM cross‐sections of nanoparticle with other mass fractions are shown in Figure S6. (G) Summary of relevant refractive indices. The effective refractive indices of P2VP microdomains containing TiO_2_‐HA/DA nanoparticles were obtained using the Bruggeman effective‐medium model [43], evaluated at nanoparticle loadings matching those used for the TEM samples (A–F). The TiO_2_ refractive index was taken from anatase and adjusted so that n(585nm)=1.9, in line with the nanoparticle datasheet [44]. Scale bars: 2 μm (A–C) and 500 nm (D–F).

Specifically, multiple, independent observations support assigning the NP‐rich lamellae to the P2VP domains. First, comparative blending experiments with three different block copolymer matrices demonstrate that selective incorporation only occurs in P2VP‐containing systems (PS‐*b*‐P2VP and P2VP‐*b*‐PMMA), while pronounced agglomeration is observed in the P2VP‐free control (PS‐*b*‐PMMA), as detailed in Section 2.1. This trend suggests that favorable interactions between TiO_2_‐HA/DA surface/shell and P2VP are necessary to prevent aggregation and enable selective incorporation [34, 45]. Second, under the TEM imaging conditions used here, the dark NPs appear within the darker lamellae. This implies that the NPs occupy the same phase that exhibits the higher elastic scattering cross‐section. This corresponds to the P2VP phase due to its nitrogen atom, which increases the effective mass–thickness contrast (Figure S8). Third, asymmetric swelling experiments provide a direct chemical assignment of the lamellae. More specifically, adding 2,4,6‐triiodophenol (TIPh) alongside the nanoparticles produces a distinct lamellar asymmetry due to its interactions with P2VP [27]. In this case, only one domain thickens (Figure S9A,B). The thickened lamellae contain nanoparticles, linking the swelling response directly to nanoparticle partitioning and assigning the nanoparticle‐rich lamellae to the P2VP domains. Fourth, the outermost shell of the concentric lamellar morphology appears as a dark layer in the pristine PS_185kDa_‐*b*‐P2VP_195kDa_ microspheres (Figure S10A) and remains dark after TiO_2_‐HA/DA incorporation (Figure S10B, $f_{\mathrm{NP}}=110\;\%$). This observation is consistent with P2VP preferentially locating at the oil‐water interface due to its greater polarity and hydrophilicity than PS. Taken together with literature reports of preferential interaction between pyridine‐containing blocks and various inorganic nanoparticle surfaces/ligands [46] and [47], these results strongly suggest that TiO_2_‐HA/DA partitions preferentially into the P2VP domains of PS_185kDa_‐*b*‐P2VP_195kDa_.

This is advantageous because the refractive index of P2VP is approximately 0.040±0.004 higher than that of PS in the 400‐700 nm wavelength range (Figure 4G). Selective filling increases the refractive index contrast in the desired direction. At intermediate loadings (i.e., $f_{\mathrm{NP}}=45\;\%$), concentric lamellar order persists in microspheres exceeding 10 μm in diameter (Figure S11). However, at loadings of $f_{\mathrm{NP}}\approx 90\;\%$, lamella wrinkling emerges, particularly in large spheres (Figure S12A) and intensifies at higher nanoparticle loading (Figure S12B). The resulting azimuthal thickness variations broaden the reflection Bragg peak and promote incoherent scattering, leading to color desaturation.

We hypothesize that the observed wrinkling arises from a buckling‐type mechanical instability resulting from two coupled effects. First, adding TiO_2_ alters the vitrification of the P2VP microphase. This causes it to solidify at a higher chloroform concentration than the PS microphase. Second, a radial chloroform concentration gradient forms within the spheres, as chloroform diffuses outward into the surrounding water. These effects lead to the formation of a solid outer crust while the sphere's interior remains liquid‐like. As the solvent continues to diffuse outward, the inner volume shrinks. This generates compressive stress in the rigid shell, ultimately leading to buckling. The resulting wrinkling pattern propagates inward as drying progresses, becoming further stabilized by the formation of concentric lamellae. This mechanism is similar to the development of roughness in spin‐cast films, as discussed in [48]. These observations define a practical loading limit for optically useful pigments rather than the absolute thermodynamic saturation threshold of TiO incorporation.

As previously mentioned, maintaining lamellar order and preventing nanoparticle agglomeration are essential to avoid incoherent scattering. TEM cross‐sections (Figure S13A,B) show that adding *h*PS, a PS‐selective homopolymer that increases PS lamella thickness [19], improves the uniformity of the concentric lamellar microstructure and reduces NP agglomeration. In addition to redshifting the optical response by increasing the thickness of the PS layers, *h*PS appears to promote more domain‐confined nanoparticle incorporation by reducing defect‐rich regions and increasing the separation between adjacent, nanoparticle‐containing lamellae. This limits aggregation‐driven incoherent scattering.

Figure 4G summarizes the refractive indices of PS, P2VP, titania nanoparticles, and the calculated effective refractive indices of P2VP–TiO_2_‐HA/DA using the Bruggeman model (Figure S14B) [43]. No changes are expected for PS. However, at $\phi_{\mathrm{NP}}=61\;\%$, the effective refractive index of P2VP–TiO_2_‐HA/DA increases to n(400nm)=1.91 and n(589nm)=1.79. This compares to n(400nm)=1.66 and n(589nm)=1.64 for pristine P2VP. At $\phi_{\mathrm{NP}}=32\;\%$, the index enhancement is Δn=0.19 at 400 nm and Δn(589nm)=0.08. Consequently, the refractive‐index contrast $n_{P2\mathrm{VP}}/n_{\mathrm{PS}}$ increases from 1.02 to 1.10 at λ=400nm and from 1.03 to 1.08 at λ=589nm. Figure S14A shows the photonic crystal index contrast for this nanoparticle loading compared with the unloaded system.

For the subsequent optical characterization, the nanoparticle loading was limited to a mass fraction of $f_{\mathrm{NP}}=40\;\%$, corresponding to a volume fraction of $\phi_{\mathrm{NP}}=27.8\;\%$ while a higher nanoparticle content may enhance the reflectance peak, it also induces excessive lamellae shrinkage, causing a blue shift in the Bragg peak. This requires compensation via swelling additives. However, excessive swelling ratios lead macrophase separation of *h*PS_35kDa_, disrupting internal order and resulting in a significant reduction in color intensity (Figure S15).

#### 2.1.5 Effect of Titania Incorporation on Refractive Index Contrast and Structural Color

Next, we examined how TiO_2_‐HA/DA nanoparticles affect the optical properties of the resulting microparticles, which were self‐assembled at $T=60^{\circ }C$.

##### 2.1.5.1 Optical Properties

Figure 5A compares reflectance spectra of photonic pigments in the dry state with and without TiO_2_‐HA/DA nanoparticles. We exploited the selective incorporation of titania into the P2VP lamellae to enhance the refractive‐index contrast between the alternating domains. This increased the reflectance intensity, color saturation, and brightness of the photonic pigments. Consistent with this design strategy, incorporating titania increased the peak reflectance compared to reference microspheres with an identical polymer composition but without nanoparticles. The purple trace (reflectance peak around 400 nm) corresponds to microspheres prepared from PS_240kDa_‐*b*‐P2VP_296kDa_, *h*PS ($M_{w}\approx 35$ kDa), and TiO_2_‐HA/DA nanoparticles at a mass loading of $f_{\mathrm{NP}}=40\;\%$, corresponding to a nanoparticle selective volume fraction of $\phi_{\mathrm{NP}}=27.8\;\%$. At this loading, the effective refractive index of the nanoparticle‐containing lamella is n=1.77 at the peak wavelength. This corresponds to a refractive index‐contrast of 1.09 with respect to the PS layer. Nevertheless, the measured reflectance remains moderate despite the enhanced color brightness at higher TiO_2_ loadings. This is likely due to the combined influence of the limited dielectric contrast of the hybrid polymer/inorganic layers, the finite number of concentric periods, and structural disorder within the microspheres. Future work could potentially increase the dielectric contrast through selective polymer removal or additional inorganic infiltration. While these strategies could improve reflectance intensity, preserving the integrity of the onion‐like particles during post‐processing would remain a key challenge.

**FIGURE 5:**
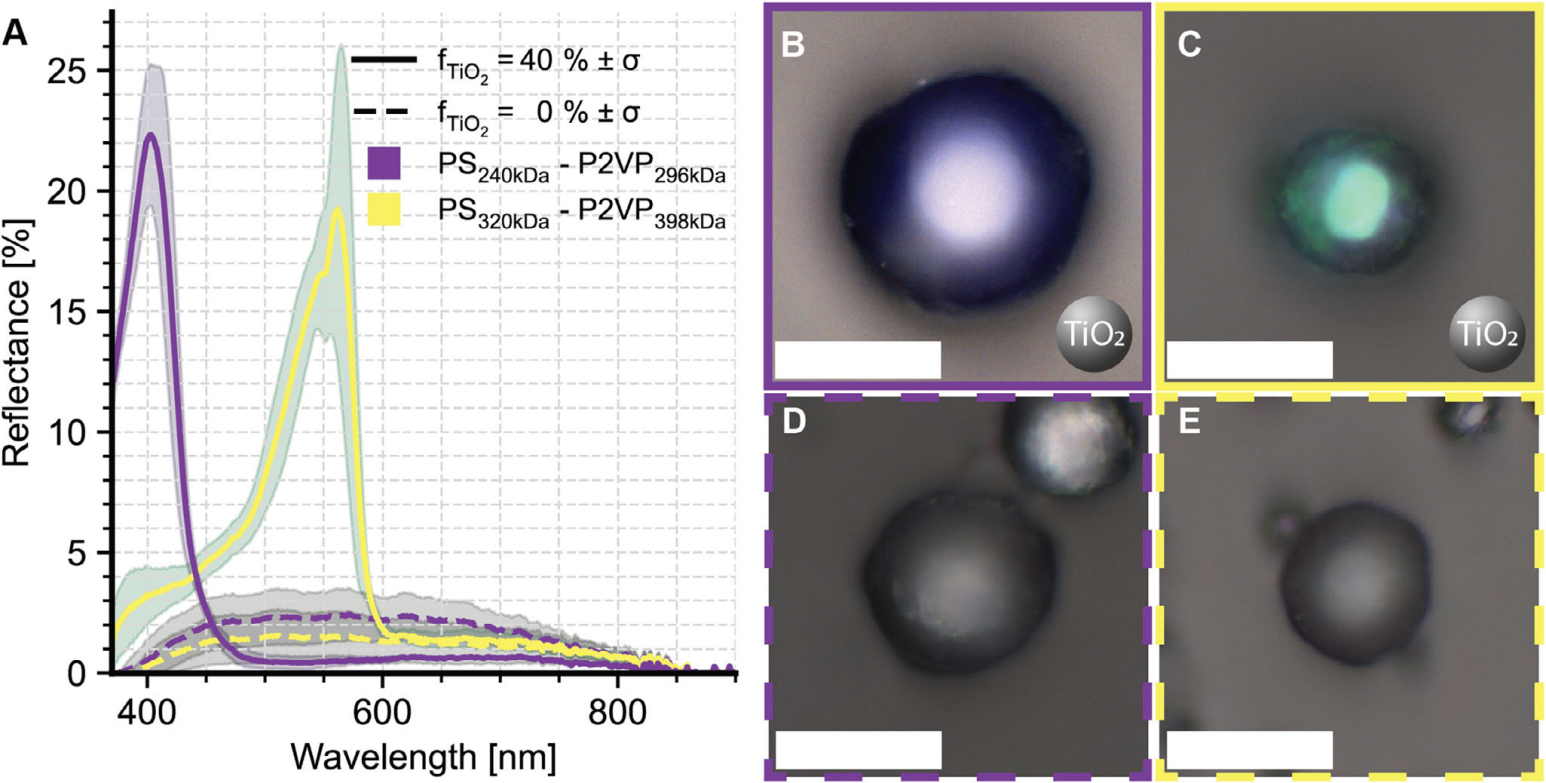
(A) Bright field optical microscopy reflectance measurements taken at 50× magnification and an NA of 0.8. Selective TiO_2_‐HA/DA loading in P2VP domains increases the refractive index contrast between lamellae, resulting in stronger constructive interference. This contributes to visibly increased color saturation and brightness. Using a block copolymer with a higher degree of polymerization yields thicker lamellae and shifts the reflectance peak to the red end of the spectrum (yellow/green curves). (B–E) show representative microspheres for the same sample sets under identical conditions. All the scale bars are 50 μm.

It should be noted that incorporating titania shifts the Bragg reflection peak through two competing contributions. First, it decreases the effective domain thicknesses $d_{P}2\mathrm{VP},\mathrm{eff}$ and $d_{\mathrm{PS}}$ (Section 2.1.2). This results in a blue shift of the reflected wavelength. Conversely, titania increases the effective refractive index $n_{P}2\mathrm{VP},\mathrm{eff}$, promoting a red‐shift of the reflection peak. To shift the photonic bandgap into the visible spectrum, *h*PS was added as a PS‐selective homopolymer sweller. The corresponding swelling volume was calculated using

(5) $$V_{\mathrm{Sweller}}=\frac{V_{\mathrm{BCP}}\;\mathrm{Block}\;I+V_{\mathrm{BCP}}\;\mathrm{Block}\;\mathrm{II}}{2}\cdot \mathrm{Swelling}\;\mathrm{Ratio}$$

Since the additives are selective swellers, the swelling volume is relative to the average pristine BCP block volume. A swelling ratio of 25 % was used. As expected for a 1D lamellar photonic structure, increasing the layer thickness (and thus the optical path length) redshifts the reflection peak according to Bragg's law [17]. The normal‐incidence peak position given by

(6) $$m\cdot \lambda_{\max}=2\cdot \left(n_{1}d_{1}+n_{2}d_{2}\right),$$

where m is an integer [49]. As previously discussed, *h*PS preferentially partitions into the PS domains and can introduce lamellar asymmetry [19]. However, in our composite pigments, this asymmetry is partially counterbalanced because TiO_2_‐HA/DA segregates into the P2VP lamellae.

Importantly, the reflectance spectrum of microspheres without nanoparticles shows no well‐defined Bragg peak. This indicates strong color desaturation and reduced luminance due to the lower refractive index contrast of 1.02 between lamellae composed of PS and P2VP alone. Concentric lamellae order is maintained in both cases, as shown in Figure S16. To achieve hues spanning the full visible wavelength range while maximizing saturation and luminance, the reflectance peak must be redshifted further. Two strategies were considered in this work: (1) increasing the degree of swelling by adding more *h*PS, and (2) using block copolymers with a higher molecular weight. Yang et al. demonstrated that combining 3‐*n*‐pentadecylphenol (PDP) and low‐molecular‐weight *h*PS enables the systematic tuning of the lamellar periodicity in PS‐*b*‐P2VP systems [19]. Dodero et al. generalized the design rule that two additives are required, with at least one binding strongly to a BCP block to overcome the entropic penalty associated with large swelling ratios [14]. However, PDP was excluded from the present study due to its low refractive index (n(589nm)=1.49) [50], and a single swelling agent (*h*PS_35kDa_) was used. This strategy, however, is constrained by an upper limit on effective swelling. At swelling ratios of ≥50% *h*PS_35kDa_, the samples exhibit signatures of excess *h*PS segregation (macrophase separation from the PS block), which prevents further homogeneous swelling of the concentric lamellae (Figure S15). This behavior was observed for all block‐copolymer molecular weights investigated: PS_185kDa_‐*b*‐P2VP_195kDa_, PS_240kDa_‐*b*‐P2VP_296kDa_, and PS_320kDa_‐*b*‐P2VP_398kDa_.

Accordingly, option (2) was pursued within the stable swelling regime. PS_320kDa_‐*b*‐P2VP_398kDa_ combined with *h*PS_35kDa_ at a swelling ratio of 25 % and $f_{\mathrm{NP}}=40\;\%$ TiO_2_‐HA/DA (corresponding to a nanoparticle volume fraction of $\phi_{\mathrm{NP}}=27.7\;\%$) produced a reflectance peak near 560 nm, as shown by the yellow spectrum in Figure 5A. Further red‐shift could potentially be achieved by selectively swelling P2VP domains with TIPh instead of PDP, as reported by Bertucci et al. [27], owing to its higher refractive index (n=1.82) [51]. Nonetheless, we do not include TIPh in this study because adding a small‐molecule swelling agent introduces additional thermodynamic and specific‐interaction effects that can alter self‐assembly in the already multicomponent PS‐*b*‐P2VP/hPS/TiO_2_ formulation, thereby expanding the parameter space beyond the scope of this work.

##### 2.1.5.2 Color Dependence of Physical Environment

Interestingly, a pronounced medium‐dependent optical response was observed when measuring PS_185kDa_‐*b*‐P2VP_195kDa_ photonic pigments with two distinct compositions in dry vs. wet environments (Figure S17): **(i)** *nanoparticle‐free pigments* and **(ii)** *nanoparticle‐loaded pigments* containing TiO_2_‐HA/DA at a mass fraction of $f_{\mathrm{NP}}=25\;\%$. In the dry state, the nanoparticle‐free pigments exhibit no pronounced reflection peak, achieving a reflectance of only ∼7%. In contrast, the nanoparticle‐loaded pigments exhibit a clear structural color, with a reflection peak at $\lambda_{\mathrm{dry}}=471.0\;\mathrm{nm}$ and a peak reflectance of 13.8%. Upon immersion in water, the two pigment types respond in opposite ways. The nanoparticle‐free pigments develop a stronger structural coloration, shifting their reflectance peak to $\lambda_{\mathrm{wet}}=526.3\;\mathrm{nm}$ and increasing their peak reflectance to 14.3%. Conversely, the reflectance peak of the nanoparticle‐loaded pigments shifts slightly to $\lambda_{\mathrm{wet}}=487.7\;\mathrm{nm}$, and the peak reflectance decreases markedly to 5.4%. These opposite trends are consistent with the micrographs (Figure S17C–F): nanoparticle‐free pigments become more colorful when wet, while the nanoparticle‐loaded pigments become less colorful.

The observed spectral changes are consistent with selective water uptake into the more polar P2VP‐containing domains. This modifies the optical path length and/or the refractive index contrast that governs Bragg‐type reflection in PS‐*b*‐P2VP‐based photonic structures. Notably, the first spectrum is acquired in the dry state, while the first in the wet state is approximately ∼30s after water was added, which is the time required to place the sample under the microscope and refocus. Consequently, the reported equilibration time of ∼3min should be interpreted as a lower bound. The spectral time series does not capture faster dynamics occurring within the first ∼30s.

The presence of TiO_2_‐HA/DA nanoparticles increases the refractive index contrast at λ=589nm from 1.027 (without ${\mathrm{TiO}}_{2}$) to 1.048 (with ${\mathrm{TiO}}_{2}$). This contributes to the distinct optical response of nanoparticle‐loaded pigments and their different absolute reflection wavelengths in wet environments. Furthermore, this optical response was reproducible across multiple independent sample batches, each containing >50 analyzed particles. Reversibility was confirmed over five wetting/drying cycles. The drying step was performed overnight, and the same samples were remeasured the following morning. These results suggest that the switching is not dominated by irreversible structural rearrangements within the experimental timeframe.

The optical response of photonic particles remained unchanged in wet environments over a pH range of 7 to 12. Since P2VP becomes strongly protonated (and thus much more hydrophilic) under acidic conditions (typically below pH≈5), the weak pH dependence in this alkaline‐to‐neutral range suggests that hydration and solvent uptake dominate protonation‐driven ionization under these conditions. No comparable optical change was observed in cyclohexane. Measurements were carried out at room temperature in all media.

### 2.2 Modeling Color Response

To explain the optical response of the onion‐like composite microspheres, we used two methods: (i) a transfer‐matrix approach for an idealized planar Bragg stack and (ii) an Ewald‐sphere construction for a concentric “Bragg onion” geometry. These models were not intended to provide a complete quantitative description of the full optical response of real microspheres. Rather, they were intended to identify the dominant optical contributions, rationalize the influence of measurement geometry, and clarify the origin of deviations between simplified models and experiments. Since these models are based on different geometric and optical assumptions, they are not expected to agree quantitatively. The transfer‐matrix approach neglects curvature, angular averaging, and scattering losses associated with structural disorder. In contrast, the Ewald‐sphere construction relies on a weak‐scattering approximation and cannot fully capture the optical response at high polar angles. These limitations motivate the future implementation of more complete wave‐optical approaches, such as Mie scattering calculations, to account for scattering contributions arising from the sphere's finite morphology.

#### 2.2.1 Bright‐Field Microscopy and Spectral Measurement Considerations

Bright‐field optical microscopy was used to evaluate the colorimetric quality of the fabricated BCP photonic pigments. The following factors must be considered when acquiring images and taking spectral measurements: sphere size, magnification, objective numerical aperture (NA), reflectance peak position, focal point, and substrate. Notably, the reflectance peak broadened toward the blue at higher NA (Figure S18). Maiwald et al. [52] explained this phenomenon using the Ewald‐sphere construction. They assumed a weak scattering limit with a refractive index contrast close to 1, such that multiple scattering was suppressed and the Born series could be truncated after the first term [53]. This leads to the reciprocal‐space diffraction condition $k_{s}-k_{i}\;\;=\;\;G$, where $k_{i}$ and $k_{s}$ are the incident and scattered wave vectors, respectively. G is any reciprocal vector for which the structure factor of the stratified sphere is non‐zero. For an infinitely large sphere, the structure factor diverges on concentric spheres with radii that are an integer multiple of $G_{1}\;=\;2\pi /\left(n_{\mathrm{eff}}L\right)$, where L is the periodicity of adjacent shells and $n_{\mathrm{eff}}$ is the sphere's effective refractive index. The real‐ and reciprocal‐space schematics for NAs of 0.22 and 0.80 are shown in Figure 6 (A,B) and Figure 6C,D, respectively. For elastic scattering ($|k_{i}\left|=\right|k_{s}|=2\pi /\lambda$), this is equivalent to Bragg's law,

(7) $$m\cdot \lambda =2\cdot n_{\mathrm{eff}}\cdot L\cdot \cos\left(\frac{\gamma }{2}\right)\;,$$

where γ is the “tail‐tail” angle between incoming and outgoing wave vectors, and m is an integer. This condition establishes strict selection rules for scattering angles at a given optical path length ($n_{\mathrm{eff}}\;\cdot \;L$) and wavelength. Therefore, the wavelength range of the collected signal depends strongly on the illumination and collection NA [15]. Two distinct cases can be distinguished.

**FIGURE 6:**
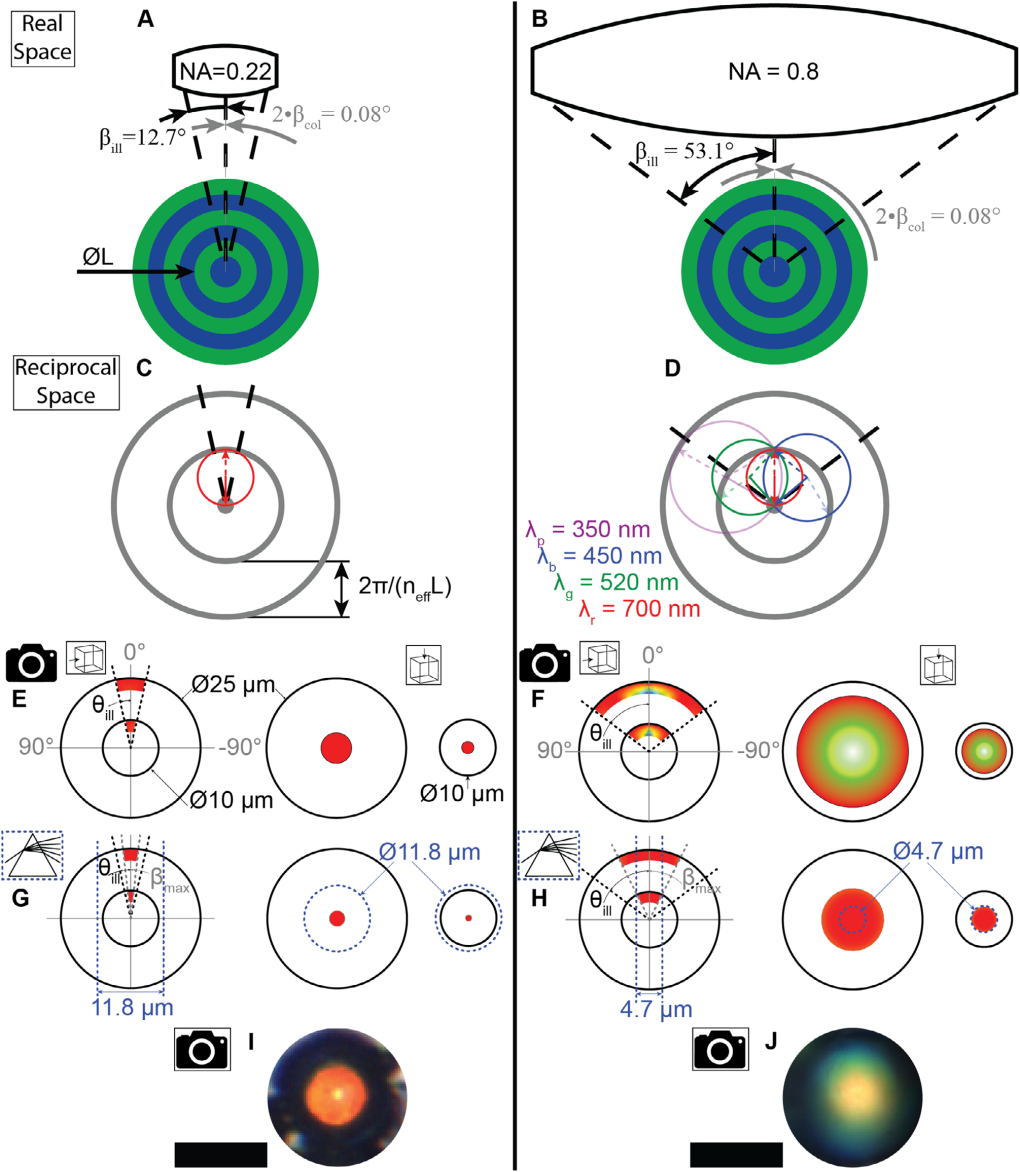
Fiber spectroscopy and camera imaging of a photonic microparticle at NA=0.22 (left column) and NA=0.8 (right column). (A,B): Side‐view cross‐sections illustrating illumination cones (half‐angle $\theta_{\mathrm{ill}}$), which equals the objective acceptance angle for the camera image, and the tiny fiber collection cone (half‐angle $\theta_{\mathrm{col}}$). (C,D): Ewald‐sphere construction [15, 52]. The gray circles represent the spherical Bragg reflexes with radius $G_{m}\;=\;m\cdot G_{1}$ (here $G_{1}\;=\;2\pi /\left(n_{\mathrm{eff}}\;\cdot \;L_{1}\right)\;=\;2\pi /350\;nm$). An Ewald sphere with radius 2π/λ (colored circles) is placed such that the incoming wave vector points toward the origin (solid arrow). Bragg scattering thus occurs at wave vectors that point to the intersection of the Ewald and a Bragg sphere (dashed arrow). Although the second‐order Bragg peak ($\lambda_{p}\;=\;350\;\mathrm{nm}$) lies in the UV, its spectral width can extend into the visible range. (E,F): Projected fiber collection spots (dashed circle) and the red spot, where Bragg reflection can occur within the NA when the fiber‐spectrometer is used (11.8 μm at 20×, NA=0.22; 4.7 μm at 50×, NA=0.8) for a 230μm fiber. Two typical sphere sizes (10 μm and 25 μm) are shown in side‐ and top‐view with polar color distributions. (G,H): Expected camera image color due to the Bragg condition only (not considering the Jacobian, lens efficiency, scattering efficiency, etc.). While the Bragg scattering of blue light shown in (D) is only possible from the north pole of the sphere within the NA, light of higher wavelengths can scatter at decreasing latitude. (I,J): Camera images of the same sample set at both NAs. Scale bar: 25 μm.

##### 2.2.1.1 Case 1: Image Acquisition (Camera)

The NA determines the range of illumination and collection angles in the microscope. Wave vector angles measured from the optical axis are denoted with θ. For a given objective, the illumination/collection half‐angle is given by $\theta_{\mathrm{ill}}\;=\;\sin^{-1}\;\left(\mathrm{NA}/n\right)$, where n is the refractive index of the surrounding medium (here, n=1 for air) [54]. Under bright‐field conditions, the incident and collected rays span a cone of angles. Thus, the scattering angle γ between the incoming and outgoing wave vectors varies across the sphere's surface. For small NA, the collection is dominated by external reflection (at the interface between the sphere and the exterior), that is p=0 [55], which is strongest at the Bragg condition. At wavelengths far away from the Bragg resonance, only 5% of the light is scattered due to Fresnel reflection between air and the effective medium of the stratified sphere. Figure 6G illustrates the expected red color appearance for small NA=0.22 (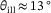) considering Bragg reflection only. β is the polar angle on the sphere's surface with the north pole as 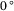 aligned with the optical axis. We, indeed see a red disk at the center of the sphere in the corresponding camera image in Figure 6I.

The camera generally captures all reflection angles that satisfy the condition $0\;\leq \;\gamma \;\leq \;2\theta_{\mathrm{ill}}$. For higher NA=0.8 (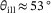), the Bragg argument leads to a peak broadening in the center of the sphere, where rays from oblique angles are also reflected into the collection aperture, satisfying the Bragg condition for smaller wavelengths. At smaller latitudes, that is with increasing polar angle β, the scattering angle γ is restricted by $\beta +\gamma /2\;\leq \;\theta_{\mathrm{ill}}$, thereby decreasing the spectral range of the reflection at lower wavelengths due to the Bragg condition (Equation 6). The expected color appearance due to the spectral range is qualitatively illustrated in Figure 6H. This does not consider the 3D nature of possible scattering events. The number of rays, for example, scales with the sin(θ) Jacobian, the lens efficiency, and the area cosine. Further, the illumination angle θ need not be coplanar with the surface polar angle β. Taking all these effects into account, we would expect an ever‐decreasing number of red rays that are reflected into the aperture at β close to the $\theta_{\mathrm{ill}}$, and thus a low intensity. However, this does still not explain why the observed color at the rim of the sphere in Figure 6J is blue.

At larger angles, where the p=0 Bragg reflection decreases, we need to consider p>1 rainbow‐like reflections from the back of the sphere [56] that scatter predominantly blue rays into the high‐NA objective. Blue rays only satisfy the Bragg condition at very large γ, which means most rays are transmitted efficiently into the sphere. The blue rays are then reflected at the back as the effective index of the sphere is slightly above 1.5, so that the focal spot is close to the sphere surface and the internal reflection angle can reach up to 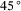, satisfying the total internal reflection condition. Finally, a considerable number of blue rays exit the sphere on the other side and are refracted into the high‐NA objective. Note that we do not observe rainbow caustics in this scenario, as the sphere is illuminated by a high‐NA beam. The observed camera image of the sphere in Figure 6J can, therefore, be qualitatively understood as the combined red to green ray p=0 Bragg reflection at larger latitudes, and the blue ray p=2 Fresnel reflection that plays a dominant role at polar angles close to, and even beyond, the NA opening angle.

##### 2.2.1.2 Case 2: Spectral Measurement (Fiber Coupling)

In fiber‐coupled spectroscopy, the recorded spectrum is an average of the imaged sample region projected onto the fiber core. The whole sphere is illuminated from the far‐field with θ spanning the NA‐limited illumination cone. The fiber is placed in the image plane of the objective, such that it captures a β‐range around the north pole of the sphere. This is achieved through reciprocal illumination through the core and by positioning the spot in the center of the sphere.

The corresponding effective collection spot diameter in the sample plane $d_{\text{disk}}$ was experimentally determined $d_{\text{disk}}$ from NA‐dependent measurements (Figure S19). For the estimates used in this work, the approximation

(8) $$d_{\text{disk}}\approx \frac{d_{\text{core}}}{M},$$

where M is the objective magnification, is sufficiently accurate.

The corresponding collection‐cone half‐angle in object space can be estimated geometrically as

(9) $$\theta_{\text{col}}\approx \arctan\;\left(\frac{d_{\text{disk}}}{2f_{\text{obj}}}\right).$$

For an infinity‐corrected microscope ($f_{\text{tube}}\;=\;165\;\mathrm{mm}$), $f_{\text{obj}}\;=\;f_{\text{tube}}/M$. Substituting $d_{\text{disk}}\;=\;d_{\text{core}}/M$ and $f_{\text{obj}}\;=\;f_{\text{tube}}/M$ cancels the magnification, yielding

(10) $$\theta_{\text{col}}\approx \arctan\;\left(\frac{d_{\text{core}}}{2f_{\text{tube}}}\right)\approx 0.04^{\circ }.$$

This corresponds to an effective collection numerical aperture

(11) $${\mathrm{NA}}_{\text{col}}\approx n\sin\theta_{\text{col}}\approx 7\times 10^{-4}\;\left(n=1\right).$$

This is orders of magnitude smaller than the NA of typical microscope objectives. Therefore, the detected signal is limited by the fiber‐coupling geometry rather than the objective NA. Consequently, the spectrometer predominantly collects near‐axial (near‐normal) reflected light.

Consequently, θ≈γ in the experiment, and most of the p=0 Bragg‐scattered light emerges from a position at β≈γ/2 on the sphere. The integrated scattering angles (and therefore the frequencies by Equation 6) are, therefore, limited by both the NA and the image spot of the fiber core. As illustrated in Figure 6E, the image spot (dashed circle) does not impose an additional limit. The spectrum shows the red Bragg peak from the center of the sphere. For large NA and large spheres (Figure 6F), the fiber spot limits the scattered frequencies, allowing for a blue‐shift, which, however, is less pronounced than expected from NA‐limited Bragg scattering alone (Figure S18).

To capture the total far‐field reflectance expected from the Laue condition and ensure that the measurement corresponds precisely to the sphere without collecting light from the surrounding area, which would reduce the reflectance, the collection disk must match the sphere's cross‐sectional area. This is demonstrated for the 10 μm sphere in Figure 6E on the right. Nevertheless, the set of excited, and therefore detectable, wavelengths depends on the illumination NA.

#### 2.2.2 Transfer Matrix Spectra Fitting

We evaluated the ability of a simple, one‐dimensional optical model to capture the color response of the fabricated, nanoparticle‐loaded photonic microspheres. Specifically, we correlated the reflectance spectrum of an individual PS_240kDa_‐*b*‐P2VP_296kDa_ sphere containing $\phi_{\mathrm{NP}}\;=\;18.2\;\%$ TiO_2_‐HA/DA with its internal lamellar morphology determined by FIB–SEM. The sphere exhibits a reflectance maximum at $\lambda_{\max}\;=\;453$ nm, with a peak height of $R_{\max}\;=\;26\;\%$ (Figure S20A, red spectrum). A FIB–SEM cross‐section (Figure S20B) of the same particle reveals an ordered lamellar stack near the surface. The lamellar periodicity is L=139.5 nm, corresponding to symmetric lamellar thicknesses of d=L/2. $N_{\mathrm{layers}}\;=\;66$ continuous lamellae can be counted from the surface to the first edge dislocation. This marks the onset of disordered/disrupted domains (Figure S20B). Using these geometric parameters and the wavelength‐dependent effective refractive indices obtained via the Bruggeman effective‐medium approximation (Figure 4), a transfer‐matrix calculation for a distributed Bragg mirror (DBR) [49] reproduces the presence and approximate position of the stop band (Figure S20A. The blue spectrum with $\lambda_{\max}\;=\;464$ nm suggests that the dominant length scale determining the spectral position is the near‐surface lamellar periodicity and the finite number of ordered layers. The remaining blueshift of the measured peak relative to the normal‐incidence transfer‐matrix prediction (i.e., 453 vs. 464 nm) is consistent with the high‐NA measurement geometry discussed above (Section 2.2.1). In other words, the experimentally collected spectrum is an angularly weighted average of a broad distribution of incidence and collection angles not included in the present calculation. However, the much lower experimental peak reflectance (26 % vs. the predicted 94 %) suggests that intensity is limited not primarily by the coherent DBR interference condition but by additional loss channels. A major loss channel is incoherent scattering from defects and domain boundaries within the near‐surface stack. Introducing a constant effective extinction coefficient (k=0.045) and making a slight adjustment to the periodicity (L=136.3 nm; $N_{\mathrm{layers}}=66$) yields a spectrum that more closely matches the measured peak position and magnitude (Figure S20A, purple spectrum). The effective extinction coefficient should not be interpreted here as an Ohmic loss, but rather as a phenomenological parameter that captures incoherent scattering losses that suppress the specular reflectance. Overall, the DBR transfer‐matrix model provides a useful first‐order description of the spectral location of the stop band. However, quantitatively predicting peak heights and the observed blue shift requires an explicit treatment of angular averaging, curvature, and scattering losses. Thus, the improved agreement obtained by introducing an effective extinction coefficient should therefore be interpreted as a phenomenological approach to account for incoherent scattering from defects and domain boundaries, rather than as a true material absorption term.

## 3 Conclusions

We have developed a composition and processing strategy to improve the dry‐state optical response of non‐iridescent block copolymer photonic pigments. This strategy combines refractive index engineering with control over structural order and scattering. A stratified architecture with a high‐refractive index contrast is obtained by selectively loading ligand‐functionalized TiO_2_ nanoparticles into the P2VP‐rich lamellae of onion‐like PS‐*b*‐P2VP microspheres. These periodic hybrid arrangements generate dry‐state Bragg reflections and corresponding structural colors that are not observed for nanoparticle‐free references under the same conditions, while retaining the angle‐robust appearance characteristic of concentric multilayer spheres.

A key finding is that titania incorporation remains efficient across the investigated composition range, enabling selective nanoparticle loading without apparent macrophase separation. However, the addition of nanoparticles couples optical gains to structural changes, with increasing the TiO_2_ content reducing lamellar periodicity and inducing disorder at higher concentrations. These structural changes link refractive index enhancement to a blue‐shift and to potential scattering‐driven desaturation when order is not maintained. These results emphasize that maximizing pigment performance requires balancing index contrast and defect‐limited scattering rather than focusing solely on contrast.

Crucially, we identify elevated‐temperature self‐assembly as an effective route to improve this balance. By promoting chain mobility during solvent removal, this processing condition reduces kinetic trapping and improves the continuity of the concentric lamellae at high nanoparticle loading. This results in more clearly defined Bragg reflections and visibly improved color appearance in the particle dry state. Building on this, we present a practical tuning concept for accessing the full visible range, as hue control requires accounting for competing optical and structural effects. Indeed, titania incorporation introduces periodicity shrinkage (blue‐shifting) vs. index increase (red‐shifting). Nevertheless, combining control over block copolymer molecular weight with selective homopolymer swelling shifts the stop band across the visible spectrum while retaining the brightness benefit of titania.

Finally, we clarify how the measured color depends on particle geometry and measurement configuration. Using a simple optical modeling framework, we explain the experimentally observed blue‐shift and peak broadening at higher objective numerical apertures. We also link microscope illumination and collection geometry to the set of excited and collected Bragg reflections, as well as to the characteristic color gradients across individual spheres. Transfer‐matrix calculations provide a first‐order description for the reflectance spectrum of a single sphere with its near‐surface multilayer geometry. The remaining mismatch (i.e., peak shift and reduced intensity) is consistent with angular averaging under high‐NA collection and additional loss channels dominated by incoherent scattering. Our findings suggest that it is essential to report and control the measurement NA and the collection geometry, as these parameters can systematically bias the peak position and apparent color quality. Moving forward, a Mie theory description of stratified spheres will quantitatively predict scattering intensities and spatial color distributions. This is expected to allow us to further optimize blue, green, and red pigment designs for maximum saturation and brightness.

## 4 Experimental Section

### 4.1 Materials

The following block‐copolymers were purchased from Polymer Source: PS_185kDa_‐*b*‐P2VP_195kDa_, PS_240kDa_‐*b*‐P2VP_296kDa_, and PS_320kDa_‐*b*‐P2VP_398kDa_, as well as PS_235kDa_‐*b*‐PMMA_263kDa_ and P2VP_235kDa_‐*b*‐PMMA_220kDa_. The subscripts indicate the number‐average molecular weight of the blocks. The polydispersity indices, defined as $D_{M}=M_{w}/M_{n}$, were 1.05 for PS_185kDa_‐*b*‐P2VP_195kDa_, 1.14 for PS_240kDa_‐*b*‐P2VP_296kDa_, 1.10 for PS_320kDa_‐*b*‐P2VP_398kDa_, 1.18 for PS_235kDa_‐*b*‐PMMA_263kDa_, and 1.17 for P2VP_235kDa_‐*b*‐PMMA_220kDa_.

Homopolymer polystyrene (*h*PS, $M_{w}\approx 35\;\mathrm{kg}\;{\mathrm{mol}}^{-1}$, Sigma–Aldrich) was used as a selective swelling agent for PS‐rich domains. Poly(vinyl alcohol) (PVA, $M_{n}=13\text{--}23\;\mathrm{kg}\;{\mathrm{mol}}^{-1}$, ∼90% hydrolyzed, Sigma–Aldrich) was used as the emulsifying surfactant in the aqueous continuous phase. This was done to suppress droplet coalescence and maintain the spherical confinement during solvent evaporation. Additionally, the adsorbed PVA layer at the droplet interface is expected to influence solvent extraction kinetics and thereby affect the self‐assembly process indirectly. Specifically, favorable interactions between PVA/water and the more polar P2VP block promote preferential localization of P2VP at the droplet interface. This favors radial lamellar orientation and the formation of concentric, onion‐like morphologies, with P2VP forming the outermost shell. Potassium chloride (KCl, ≥99%, Fisher Scientific) was added to the continuous phase at a concentration of 0.25M during solvent evaporation.

Titanium dioxide nanoparticles (TiO_2_) functionalized with hexylamine and dodecylphosphonic acid (TiO_2_‐HA/DA) were purchased from PlasmaChem GmbH and used as received. According to the supplier, the primary particle diameter ranges from 4 to 8 nm. Nitric‐acid‐functionalized TiO_2_ nanoparticles (PlasmaChem GmbH) were used as the starting material for gallic acid functionalization (see Section 4.2). All solvents were of analytical grade. N,N‐dimethylformamide (DMF) was used for ligand exchange reactions. Chloroform (CHCl_3_, amylene‐stabilized) was used for nanoparticle stock dispersions and 1,2‐dichloroethane (DCE) for block copolymer solution preparation, unless otherwise noted. Milli‐Q water (conductivity $0.055\;\mu S\;{\mathrm{cm}}^{-1}$) was used throughout.

#### 4.1.1 Densities and Volume‐Fraction Calculations

The nanoparticle volume fraction $\phi_{\mathrm{NP}}$ was computed from mass ratios using the following densities (in $g\;{\mathrm{cm}}^{-3}$): $\rho_{\mathrm{PS}}=1.06$, $\rho_{\mathrm{PMMA}}=1.18$, $\rho_{P2\mathrm{VP}}=1.153$, $\rho_{\mathrm{PDP}}=0.91$, and $\rho_{{\mathrm{TiO}}_{2}}=4.23$. The effective density of $\rho_{{\mathrm{TiO}}_{2}-\mathrm{ligand}}=2.17\;g\;{\mathrm{cm}}^{-3}$ for ligand‐functionalized nanoparticles (TiO_2_‐HA/DA) was computed via a mass‐weighted reciprocal mixing rule,

(12) $$\rho_{\mathrm{eff}}={\left(\frac{w_{{\mathrm{TiO}}_{2}}}{\rho_{{\mathrm{TiO}}_{2}}}+\frac{w_{\mathrm{lig}}}{\rho_{\mathrm{lig}}}\right)}^{-1},$$

using the weight fractions $w_{{\mathrm{TiO}}_{2}}=0.703$, $w_{\mathrm{lig}}=0.297$ (determined by TGA), $\rho_{{\mathrm{TiO}}_{2}}=4.23\;g\;{\mathrm{cm}}^{-3}$, and $\rho_{\mathrm{lig}}=1.01\;g\;{\mathrm{cm}}^{-3}$ (the ligand density was assumed to be comparable to dodecylphosphonic acid). All definitions of nanoparticle mass fraction $f_{\mathrm{NP}}$ and swelling ratios are provided in Equation 2 and Equation 5, respectively.

#### 4.1.2 Optical Constants and Interaction Parameters

The refractive indices of PS and P2VP were obtained from ellipsometric data in Dodero et al. [14]. The refractive indices of water and anatase titania nanoparticles were obtained from the works of [57] and [26], respectively. The Flory–Huggins interaction parameters used in this study were taken from the literature at 298 K: $\chi_{\mathrm{PS}-b-\mathrm{PMMA}}=0.041$ [58], $\chi_{\mathrm{PS}-b-P2\mathrm{VP}}=0.280$ [59], and $\chi_{P2\mathrm{VP}-b-\mathrm{PMMA}}=0.069$ [60]

### 4.2 Nanoparticle Functionalization and Dispersion

Commercial nitric‐acid‐treated TiO_2_ nanoparticles (TiO_2_–HNO_3_) (PlasmaChem GmbH) were functionalized with gallic acid (GA) by adsorption/ligand exchange in an aqueous medium. Then, the nanoparticles were transferred to DMF. Fifteen milligrams of GA were dissolved in 2.0 mL of Milli‐Q water in a 4 mL glass vial by ultrasonication ($c_{\mathrm{GA}}$ = 7.5 mg mL^−^). Meanwhile, 130 mg of TiO_2_ powder was dispersed in 13 mL of Milli‐Q water in a glass vial by thorough mixing ($c_{{\mathrm{TiO}}_{2}}$ = 10 mg mL^−1^). To functionalize the particles, defined volumes of the GA solution and the TiO_2_ dispersion were combined to achieve GA:TiO_2_ mass ratios between 1:50 and 1:1000. The mixtures were left to equilibrate for 2 h at room temperature. It is expected that polyphenol will bind to TiO_2_ via inner‐sphere coordination with surface Ti sites [61]. Finally, an aliquot of the resulting dispersion was diluted by adding 3.8 mL of DMF, yielding a TiO_2_ concentration of 0.5 mg mL^−1^. Nanoparticle‐to‐ligand mass ratios $m_{\mathrm{GA}}:m_{{\mathrm{TiO}}_{2}}$ were screened from 1:1000 to 1:50 to optimize dispersion stability. Ratios outside the optimum range promoted aggregation. Thermogravimetric analysis (TGA) of TiO_2_‐GA is shown in Figure S21A. Dynamic light scattering (DLS) yielded an average hydrodynamic diameter of 13.9±4.2nm (LS Spectrometer II (LS Instruments, Switzerland)).

The commercial TiO_2_‐HA/DA nanoparticles were used as received. The TGA data are shown in Figure S21B. The residual mass was found to be 70.3 %. The difference between this residual mass, which corresponds to the inorganic TiO_2_ core, and the initial total mass is attributed to the presence of the surface‐bound ligands hexylamine and dodecylphosphonic acid.

### 4.3 Photonic Microsphere Fabrication by Emulsion Solvent Evaporation

Polymer stock solutions were prepared in DCE at a concentration of $30\;\mathrm{mg}\;{\mathrm{mL}}^{-1}$ in sealed glass vials. When used, *h*PS was added to the organic phase at a concentration of $30\;\mathrm{mg}\;{\mathrm{mL}}^{-1}$ prior to emulsification at the targeted swelling ratio (definition in Equation 5). Nanoparticles were incorporated by adding the required volume of nanoparticle stock dispersion to the block copolymer solution to obtain nanoparticle‐to‐polymer mass ratios $f_{\mathrm{NP}}=4\text{--}150\;\%$ (definition in Equation 2), adding additional DCE to obtain a polymer concentration of $10\;\mathrm{mg}\;{\mathrm{mL}}^{-1}$ and mixing for 10min. Stock dispersions were prepared at $c_{\mathrm{NP}}=13.3\;\mathrm{mg}\;{\mathrm{mL}}^{-1}$ in CHCl_3_ and sonicated for 30s.

PVA was dissolved in Milli‐Q water at $85^{\circ }C$ under stirring for 3 h to obtain solutions at $10\;\mathrm{mg}\;{\mathrm{mL}}^{-1}$ (emulsification) and $3\;\mathrm{mg}\;{\mathrm{mL}}^{-1}$ (continuous phase), and cooled to room temperature prior to use.

In a typical emulsification procedure, 250μL of the organic phase was transferred to a 4mL screw‐cap glass vial and emulsified with 2.5mL of the $10\;\mathrm{mg}\;{\mathrm{mL}}^{-1}$ PVA aqueous solution using a vortex mixer at 2000rpm for 15s. The resulting oil‐in‐water (O/W) emulsion was poured into a 5cm diameter Petri dish containing 14mL of $3\;\mathrm{mg}\;{\mathrm{mL}}^{-1}$ PVA aqueous solution supplemented with 0.25M KCl. The Petri dishes were covered to slow solvent evaporation and left in a fume hood for 48h.

Although the vortex‐assisted emulsification approach used here enables rapid and scalable particle production, it does not yield highly monodisperse droplets. In future work, narrower particle size distributions could potentially be achieved using microfluidic droplet generation or membrane emulsification approaches [62]. These approaches could be useful for improving the color performance of Bragg onion‐based colored films.

For thermally assisted self‐assembly, solvent evaporation was conducted at $60^{\circ }C$ for the full 48h period (in an oven) and the continuous phase is heated to $60^{\circ }C$ prior to use.

Particles were collected by centrifugation at 5000rpm for 15min and washed three times with Milli‐Q water. Washed particles were redispersed in water to the desired concentration of $2\;\mathrm{mg}\;{\mathrm{mL}}^{-1}$ for optical and structural characterization.

### 4.4 Optical Microscopy and Microspectroscopy

Bright‐field reflection micrographs were acquired using an Axio Scope.A1 (Zeiss, Germany) light microscope connected to a CCD camera (GS3‐U3‐28S5C‐C, FLIR Integrated Imaging Solutions Inc., Richmond, Canada). Spectral data were recorded by placing an optical fiber (OceanOptics, QP230‐1‐XRS) in a plane confocal to the image plane connected to a spectrometer (OceanOptics, QEPRO–vis‐NIR) calibrated against a dielectric mirror (ThorLabs, BB07‐E02) and illuminated with a xenon lamp (ThorLabs, SLS401). Microparticles were characterized in the dry state on a carbon tape.

Objectives with magnifications of 20× (Zeiss, EC Epiplan‐Neofluar, NA 0.22 HD DIC) and 50× (Zeiss, EC Epiplan‐Neofluar, NA 0.8 HD DIC) were employed. The collection area projected on the sample plane was calibrated (Figure S19) and fitted as

$$d_{\mathrm{disk}}=\frac{286.89\;\mu m}{M^{1.08}},\;R^{2}=1.00,$$

as defined in Equation 8.

### 4.5 Electron Microscopy

#### 4.5.1 Focused‐Ion‐Beam Scanning Electron Microscopy (FIB‐SEM)

Microsphere internal structure and nanoparticle spatial distribution were investigated by FIB–SEM using a Thermo Scientific Scios 2 DualBeam FIB‐SEM (FEI, Eindhoven, the Netherlands). Particle suspensions were drop‐cast onto aluminum stubs with conductive carbon tape, dried overnight, and coated with a 4nm Au layer (Cressington 208HR, UK). Half‐sphere cross‐sections were milled with a ${\mathrm{Ga}}^{+}$ ion beam at 30kV with currents between 50 pA and 3 nA, and the cut face was imaged at 5kV and a current of 100 pA using the built‐in SEM Everhart–Thornley (ETD, secondary electrons) and in‐lens T1 (A + B composite mode, back‐scattered electrons) detectors.

#### 4.5.2 TEM Sample Preparation: Resin Embedding and Ultrathin Cross‐Sections of Microspheres

Microsphere/NP samples were collected by centrifugation (10 000 rpm), washed three times with water, and dried under vacuum overnight. The dried particles were then transferred into BEEM capsules.

##### 4.5.2.1 Embedding in EpoFix epoxy resin

EpoFix cold‐setting embedding resin (Electron Microscopy Sciences) was used for plastic embedding. The resin and hardener were mixed at a 25:3 (w/w) ratio in a closed 15 mL polypropylene tube to minimize humidity exposure and stirred until homogeneous. To each BEEM capsule containing the microsphere/NP pellet, 1.0 mL of freshly prepared resin was added without resuspending the pellet. Capsules were kept closed during curing, and polymerization was carried out overnight at room temperature.

##### 4.5.2.2 Trimming, Sectioning, and Grid Preparation

Polymerized blocks were trimmed using a razor blade. Ultrathin sections (70 nm) were obtained using a Reichert–Jung Ultracut E ultramicrotome equipped with an Ultra 45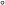 diamond knife (Diatome AG, Nidau, Switzerland). Sections were collected on formvar/carbon‐coated copper slot grids (S162‐5, Plano EM, Wetzlar, Germany) and imaged using a Veleta digital camera (Olympus) on an FEI Tecnai Spirit transmission electron microscope (Thermo Fisher Scientific) operated at 120 kV.

### 4.6 Ultra‐Small‐Angle X‐Ray Scattering (USAXS)

USAXS measurements were performed on the ID02 beamline at the European Synchrotron Radiation Facility (ESRF, Grenoble, France). Samples were prepared from concentrated particles (dry solids) by drop‐casting into Teflon washers and sealing with Kapton tape to form bulk circular specimens. Sample‐to‐detector distance was 10 m.

### 4.7 Thermogravimetric Analysis (TGA)

Nanoparticle loading and ligand content were quantified by thermogravimetric analysis using a Mettler‐Toledo TGA/DSC 1 STARe system. Measurements were conducted in flowing air ($80\;\mathrm{mL}\;\min^{-1}$) at a heating rate of $10\;C\;\min^{-1}$ from 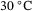 to 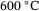 or 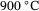 depending on the experiment.

### 4.8 Effective‐Medium Modeling of Composite Refractive Indices

To estimate effective refractive indices of nanoparticle‐filled polymer domains, effective‐medium approximations were applied. Because the targeted nanoparticle volume fractions extend well beyond the dilute limit, the Bruggeman effective‐medium approximation was consistently used [43]. The primary particle size of TiO_2_‐HA/DA (4--8nm, supplier specification) is well below visible wavelengths, supporting the optical‐homogenization assumption for refractive index estimation. The resulting wavelength‐dependent effective indices were used for refractive index contrast analysis and for comparison with measured reflectance spectra.

## Funding

This study was supported by a European Research Council (ERC) Advanced grant (PrISMoID, grant number 833895), the National Center of Competence in Research (NCCR) Bio‐Inspired Materials (grant number 51NF40‐182881) of the Swiss National Science Foundation (SNSF), and the Adolphe Merkle Foundation.

## Conflicts of Interest

The authors declare no conflicts of interest.

## Supporting information




**Supporting File**: marc70359‐sup‐0001‐SuppMat.pdf.

## Data Availability

Data supporting the findings of this study are openly  available in the Zenodo repository at https://doi.org/10.5281/zenodo.17976499.
