# Supplementary material for: Engineering Photonic Pigments From Titania–Block Copolymer Composites
Source: Macromol Rapid Commun. 2026 Jul 14;47(19):e70359. doi: 10.1002/marc.70359 (PMC13639234; doi:10.1002/marc.70359)
Supplement: Supplementary file 1 [file MARC-47-e70359-s001.pdf]

Engineering Photonic Pigments from Titania–Block Copolymer Composites

Niklas Rocca Schwarz[1,2] *Jorge Larios*[1,2] *Antoine Scalabre*[2,3] *Matthias Saba*[1,2] *Ullrich Steiner*[1,2] *Andrea Dodero*[1,2,*]

[1]Adolphe Merkle Institute, University of Fribourg, Chemin des Verdiers 4, 1700 Fribourg, Switzerland
[2]NCCR Bio-inspired Materials, University of Fribourg, Chemin des Verdiers 4, 1700 Fribourg, Switzerland
[3]Department of Chemistry, University of Fribourg, Chemin du Musée 9, 1700 Fribourg, Switzerland
E-mail: andrea.dodero@unifr.ch

Supplementary Information

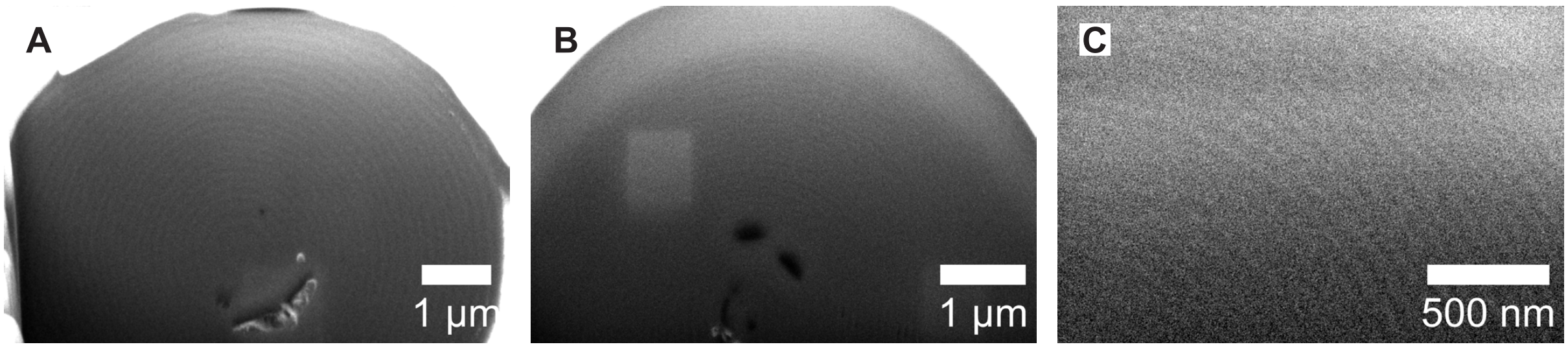

Figure S1: FIB-SEM micro-sphere cross-sections of block copolymer systems without any additives. **A** PS_{185kDa}-*b*-$P2VP_{195kDa}$ exhibits well-ordered lamellar domains with a periodicity of $L_0 \approx 133$ nm. **B** $P2VP_{235kDa}$-*b*-$PMMA_{220kDa}$ forms a similar lamellar morphology with a reduced periodicity of $L_0 \approx 97$ nm. **C** PS_{235kDa}-*b*-$PMMA_{263kDa}$ shows the smallest domain spacing among the three systems, with $L_0 \approx 78$ nm. Onion-like morphologies are observed across all block copolymer compositions, but varying molecular weights and Flory-Huggins interaction parameters yield distinct domain sizes.

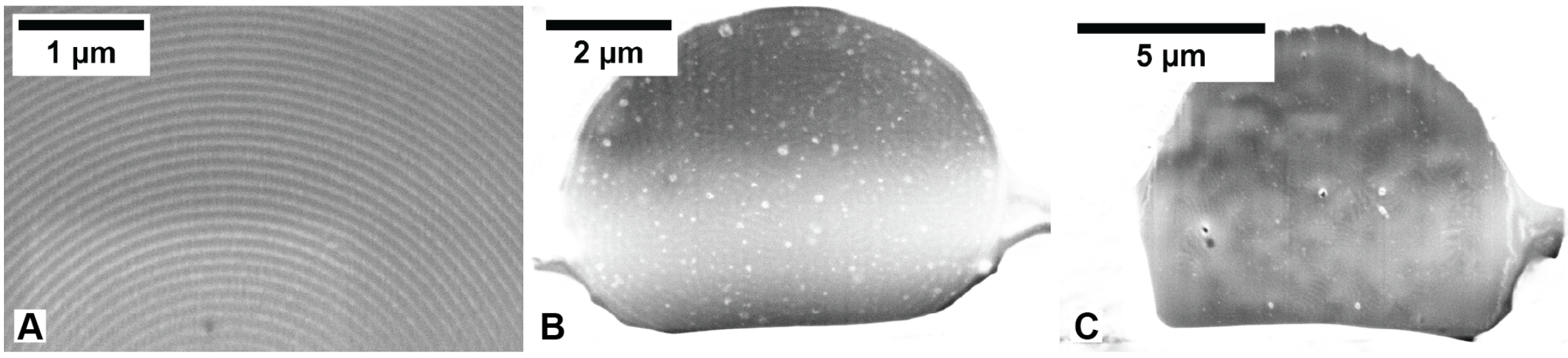

Figure S2: FIB–SEM cross-sections of microspheres with incorporated TiO_2-GA (GA = gallic acid) nanoparticles are presented for (**A**) PS_{235kDa}-*b*-$PMMA_{263kDa}$, (**B**) PS_{185kDa}-*b*-$P2VP_{195kDa}$, and (**C**) $P2VP_{235kDa}$-*b*-$PMMA_{220kDa}$. The nanoparticle mass fraction was fixed at $f_{NP} = 4\,\%$, and the ligand mass ratio was $\frac{m_{TiO_2}}{m_{GA}} \approx 100$

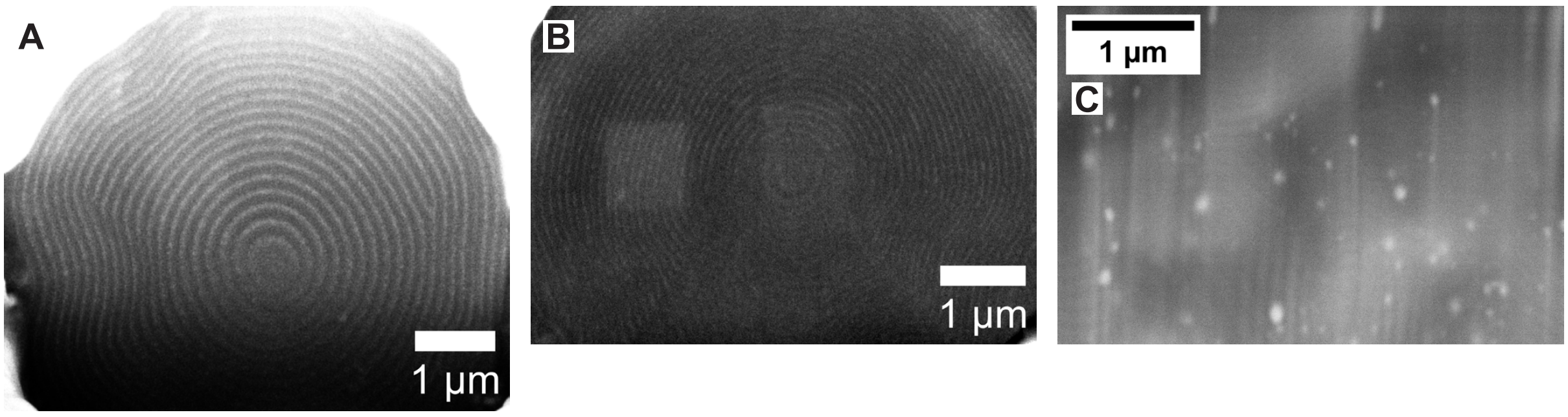

Figure S3: **A** FIB–SEM cross-section of PS_{185kDa}-b-$P2VP_{195kDa}$ with $f_{NP} = 20\,\%$ TiO_2-HA/DA. $h PS_{35kDa}$ and 3-n-pentadecylphenol are added to increase the lamellar domain thickness. Nanoparticles appear as white, grainy features selectively incorporated into alternating domains. **B** FIB–SEM cross-section of $P2VP_{235kDa}$-b-$PMMA_{220kDa}$ with $f_{NP} = 20\,\%$ TiO_2-HA/DA. The addition of 3-n-pentadecylphenol and $h PMMA_{5kDa}$ similarly increases the domain thickness, and nanoparticles segregate into alternating domains. **C** FIB–SEM cross-section of PS_{185kDa}-b-$P2VP_{195kDa}$ with $f_{NP} = 10\,\%$ TiO_2-HA/DA. White regions indicate nanoparticle agglomeration.

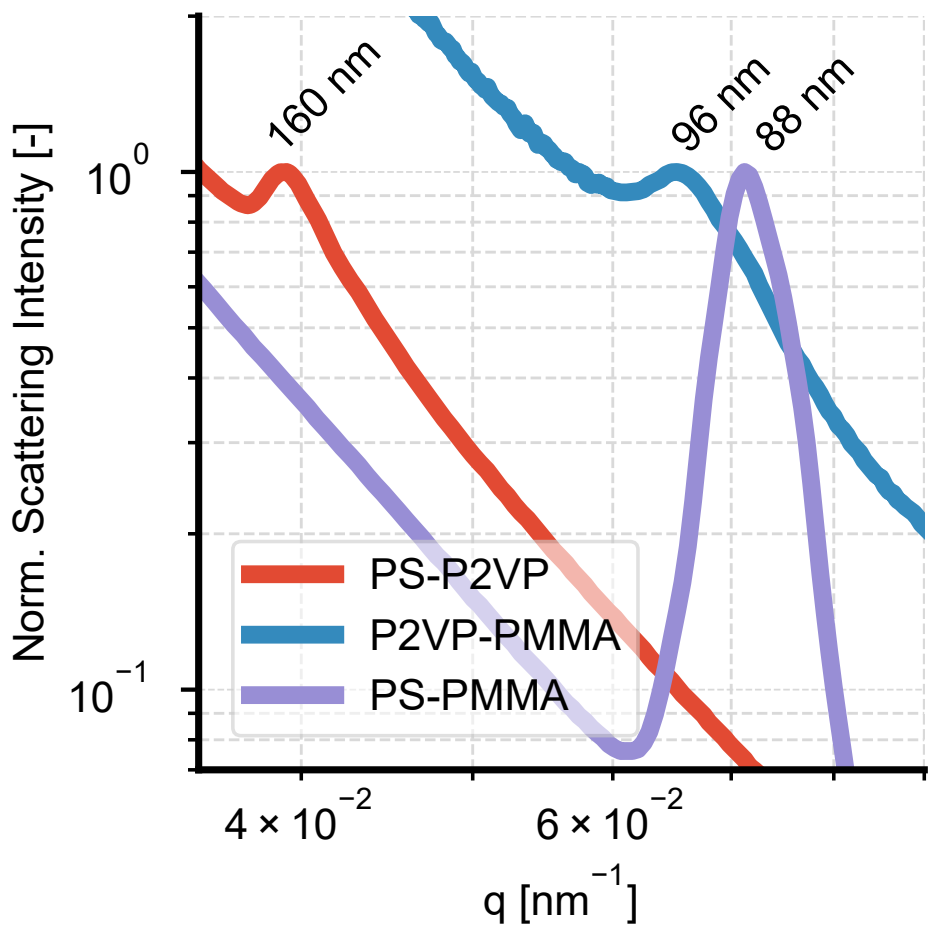

Figure S4: Scattering intensities plotted from USAXS measurements. Microspheres with concentric lamellae from PS_{185kDa}-b-$P2VP_{195kDa}$, $P2VP_{235kDa}$-b-$PMMA_{220kDa}$ and PS_{235kDa}-b-$PMMA_{263kDa}$.

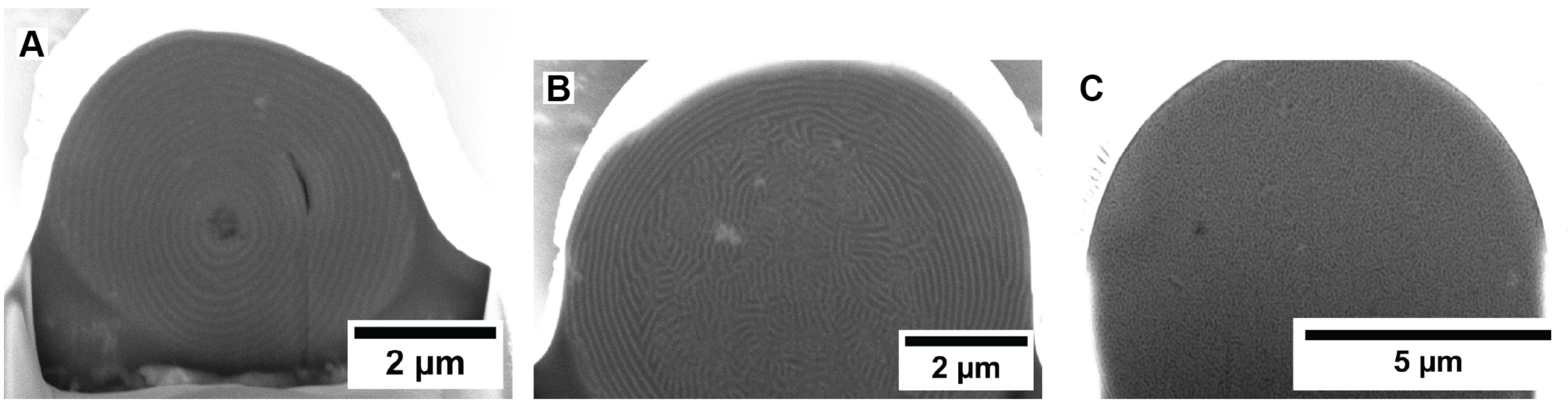

Figure S5: FIB–SEM cross-sectional micrographs of TiO_2-HA/DA incorporated in PS_{185kDa}-b-$P2VP_{195kDa}$ at nanoparticle mass fractions of $f_{NP} = 4\,\%$ **A**, $10\,\%$ **B**, and $20\,\%$ **C**. Self-assembly was performed at room temperature. Increasing nanoparticle loading leads to progressively more polycrystalline morphologies. Chloroform is the dispersed phase ,which is where the PS-b-P2VP is dissolved in.

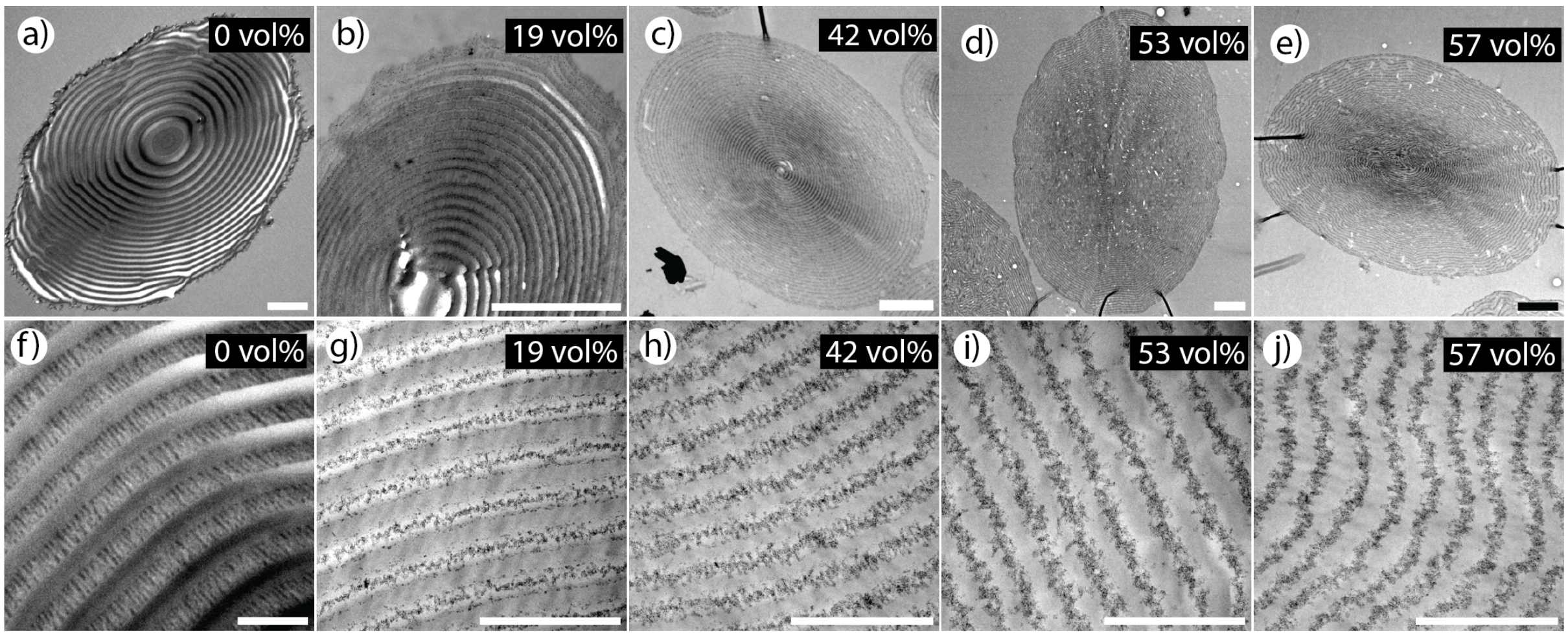

Figure S6: Transmission electron microscopy (TEM) micrographs of microsphere cross-sections containing varying volume fractions of TiO_2-HA/DA incorporated in PS_{185kDa}-b-$P2VP_{195kDa}$ as stated in percentage insets. Scale bars in a) to e) are 2 µm and in f) to j) are 500 nm.

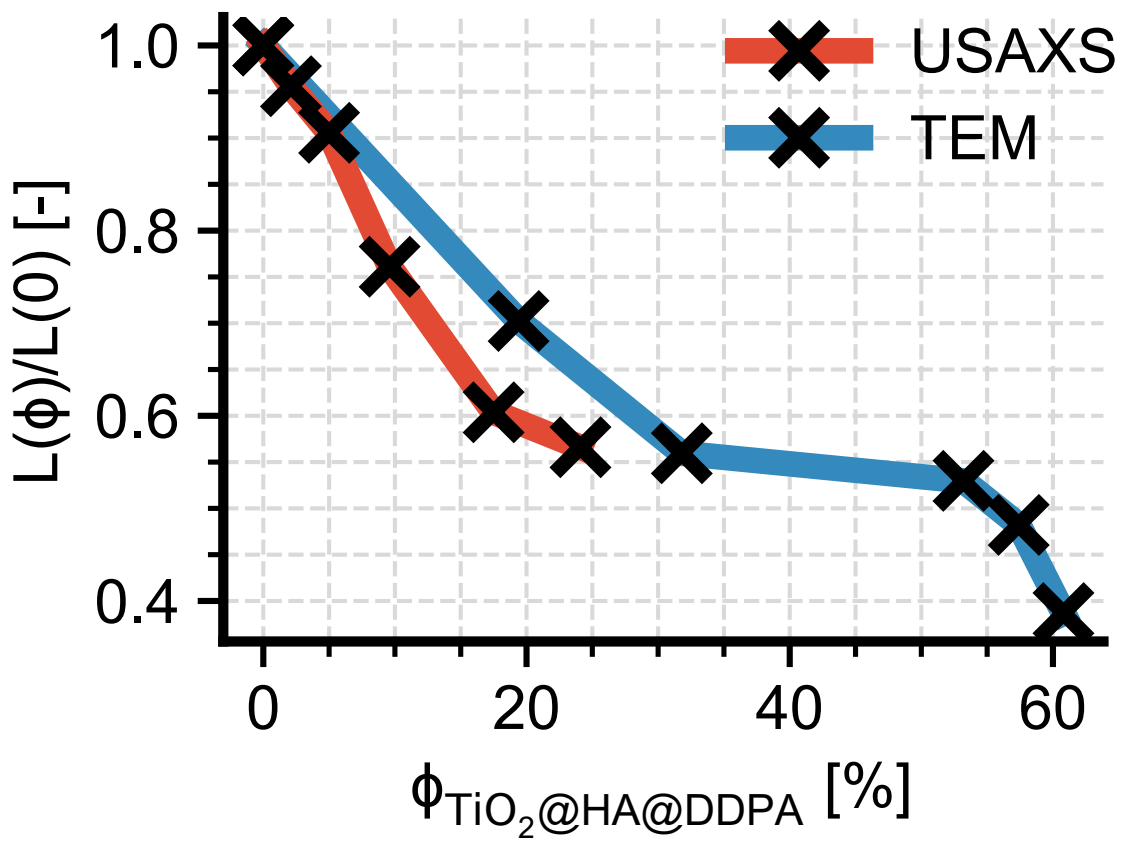

Figure S7: The lamellar periodicity L of PS_{185kDa}-b-$P2VP_{195kDa}$ microspheres is determined as a function of the titania volume fraction $\phi_{TiO_2-HA/DA}$ using two independent methods: (i) peak positions in the USAXS scattering intensity and (ii) Fourier transforms of TEM cross-section micrographs. For comparison, L is normalized to the lamellar periodicity in the absence of titania, yielding reference values of $L(0) = 159$ nm from USAXS and $L(0) = 168$ nm from TEM.

Table S1: Selected nanoparticle mass ratios f_{NP} (see Equation 2) of TiO_2-HA/DA in PS-b-P2VP block copolymers of different molecular weights, together with the corresponding nanoparticle volume fractions ϕ_{NP} in the P2VP block (see Equation 3). The table serves as a reference for the values used throughout the text and figures.

f_{NP} [%]	4.0	20.0	25.0	40.0	45.0	70.0	90.0	110.0	130.0	150.0
$\phi_{NP}(PS_{185kDa}$-b-$P2VP_{195kDa})$ [%]	4.0	17.2	20.6	29.3	31.8	42.0	48.2	53.2	57.4	60.8
$\phi_{NP}(PS_{240kDa}$-b-$P2VP_{296kDa})$ [%]	3.7	16.1	19.4	27.8	30.2	40.2	46.4	51.4	55.6	59.1
$\phi_{NP}(PS_{320kDa}$-b-$P2VP_{398kDa})$ [%]	3.7	16.1	19.3	27.7	30.1	40.2	46.3	51.3	55.5	59.0

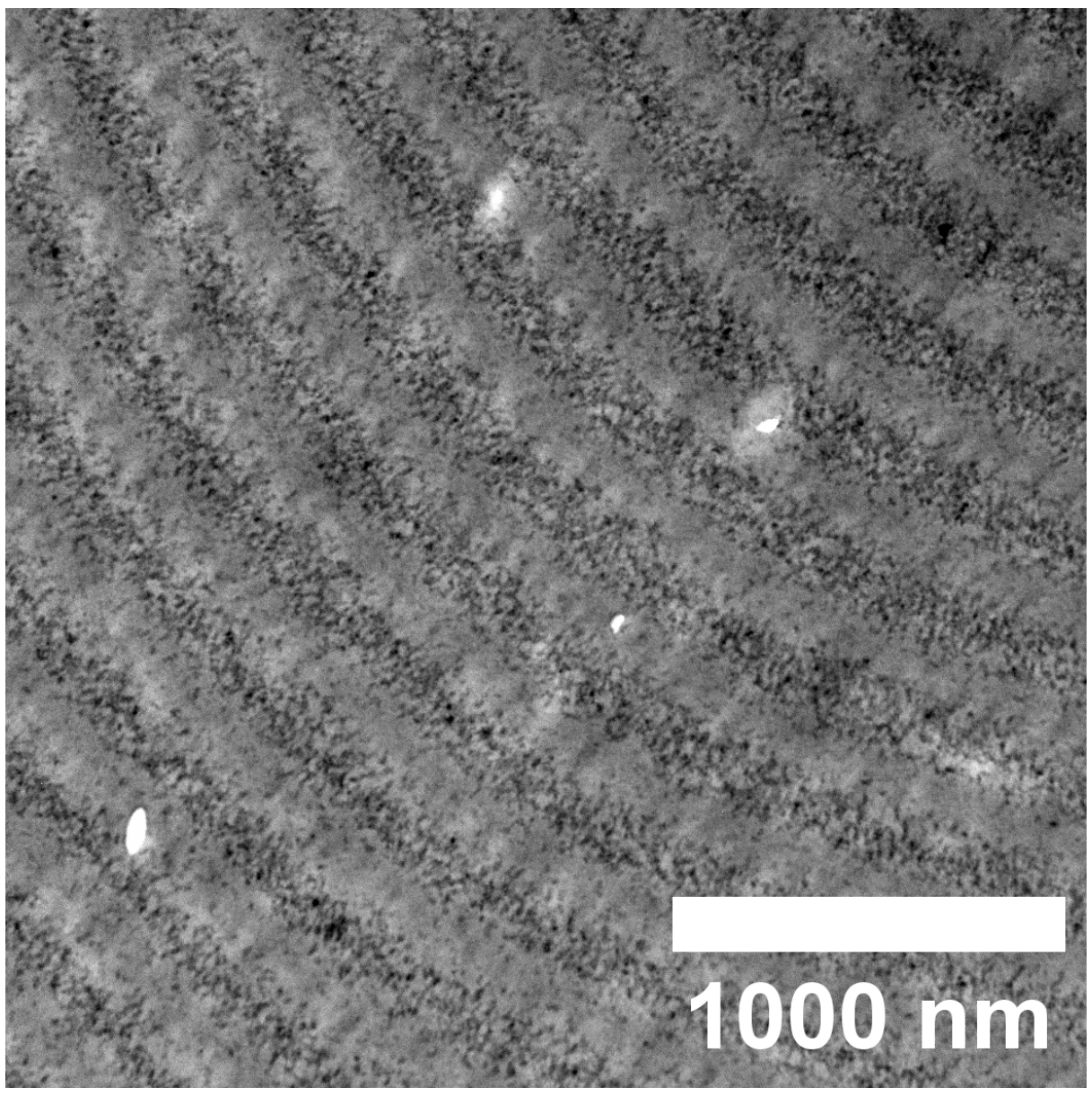

Figure S8: Transmission electron microscopy (TEM) of the same mass fraction of TiO_2-HA/DA in PS_{185kDa}-*b*-$P2VP_{195kDa}$ as shown in Figure S9 **A** and **B**, but without the addition of any other swelling molecules such as 2,4,6-triiodophenol. The theoretical volume fraction of PS is 41 %, assuming no lamellar thickness changes due to nanoparticle incorporation. This image serves as a reference for the asymmetric swelling presented in Figure S9.

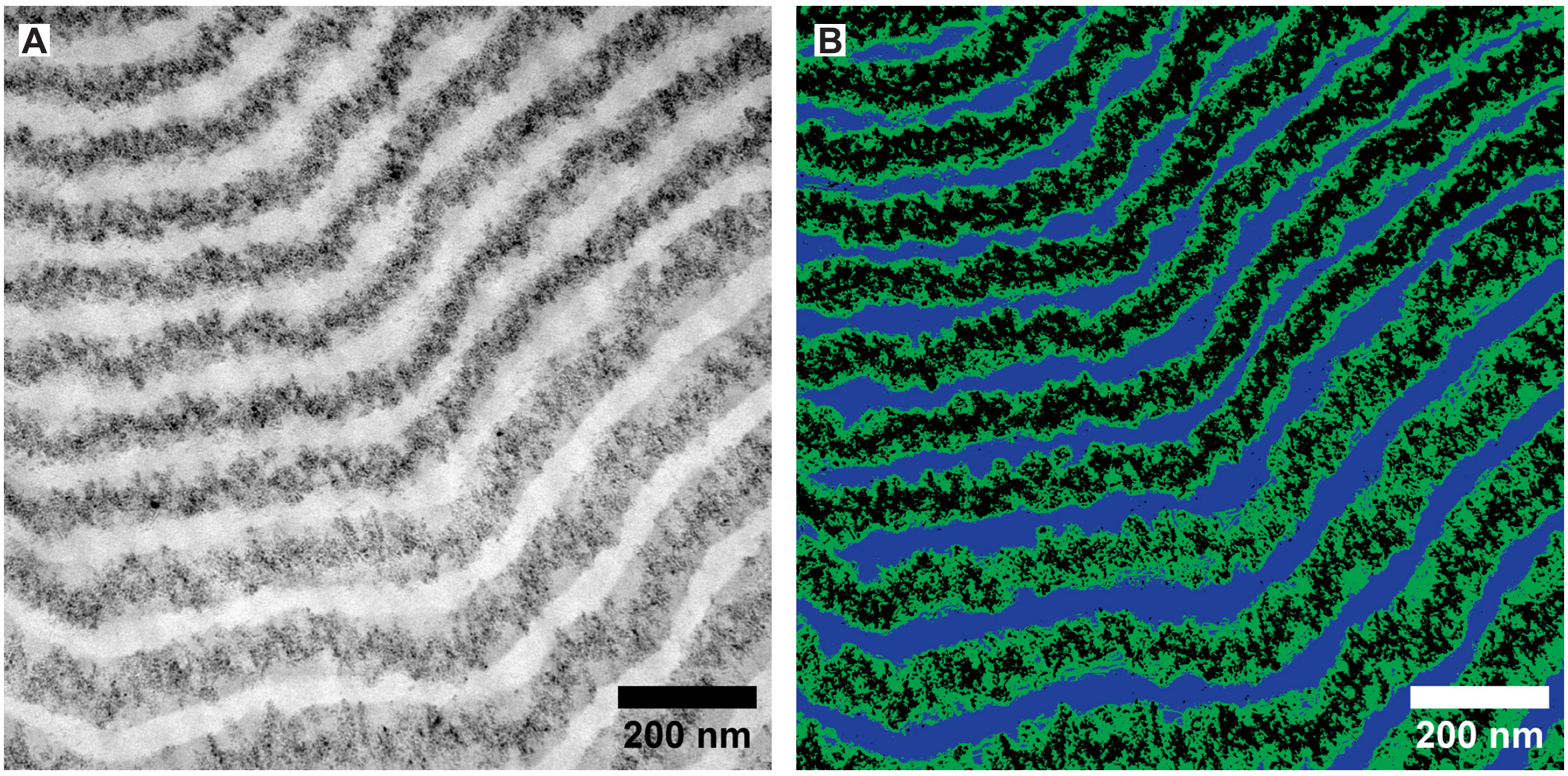

Figure S9: **A** Asymmetric swelling of PS-*b*-P2VP with 30 % swelling induced by 2,4,6-triiodophenol was used to identify which block incorporates TiO_2-HA/DA nanoparticles. A high nanoparticle loading of $f_{NP} = 62\,\%$ (corresponding to $\phi_{NP} = 33\,\%$) was introduced into PS_{185kDa}-*b*-$P2VP_{195kDa}$. TEM micrographs reveal the resulting lamellar morphology and nanoparticle distribution; compare with **Figure S8** for an unswollen reference of identical composition. **B** Segmentation of polymer blocks and nanoparticles was performed using thresholding combined with machine-learning classification. The blue region occupies 35 % of the area, compared with the theoretical PS volume fraction of 27 %, indicating assignment of blue to PS and green to P2VP. Deviations arise from imperfect swelling and lamellar thickness variations induced by nanoparticle incorporation. The predominance of nanoparticles in the green domain confirms selective interaction of TiO_2-HA/DA with P2VP in PS-*b*-P2VP.

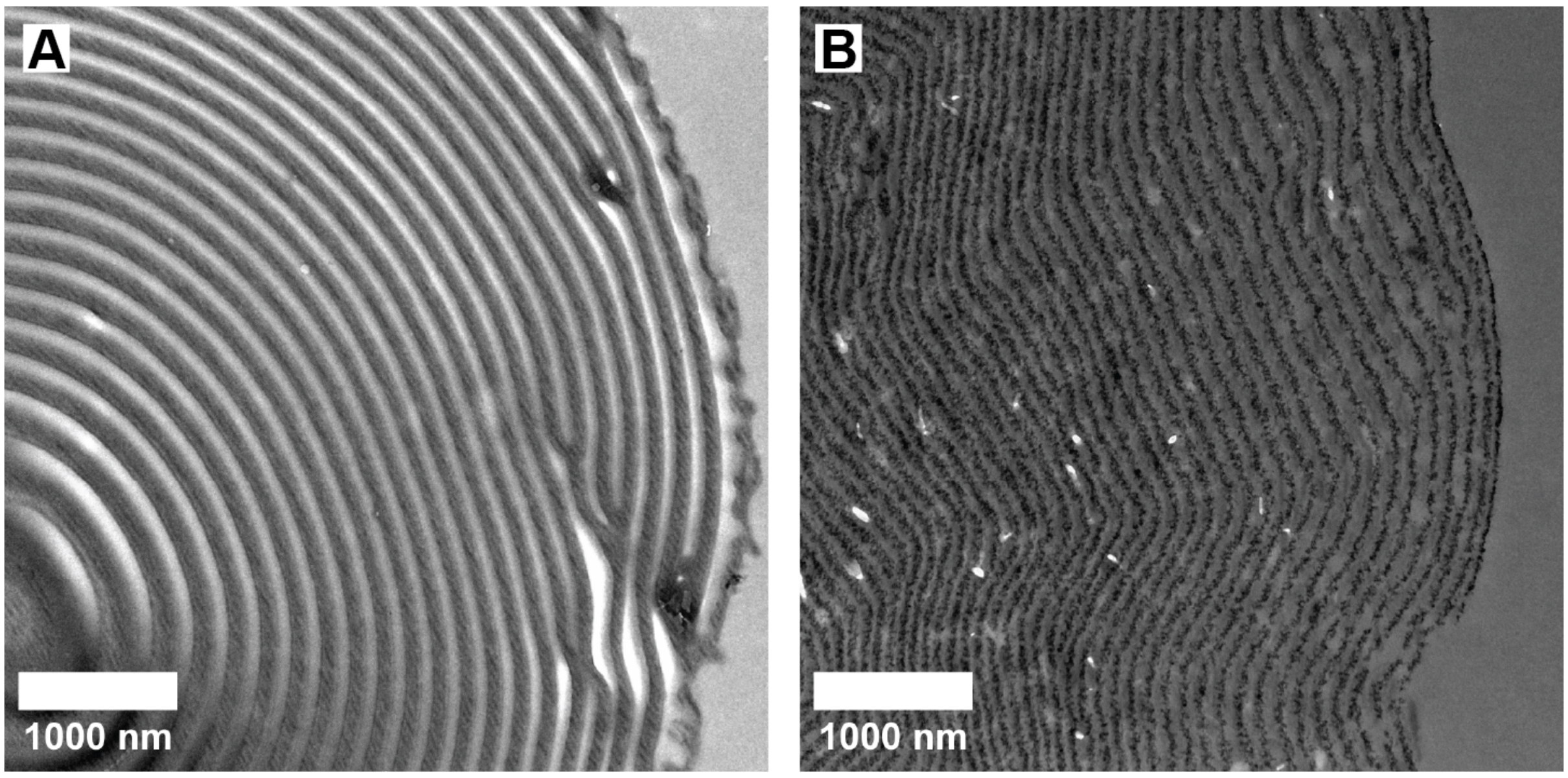

Figure S10: TEM microsphere cross-section micrographs of PS_{185kDa}-b-$P2VP_{195kDa}$. In both **A** and **B**, the outermost layer is the darker one corresponding to P2VP. **B** additionally contains $f_{NP} = 110\,\%$. The nanoparticles are also located in the darker layer, indicating their presence in P2VP.

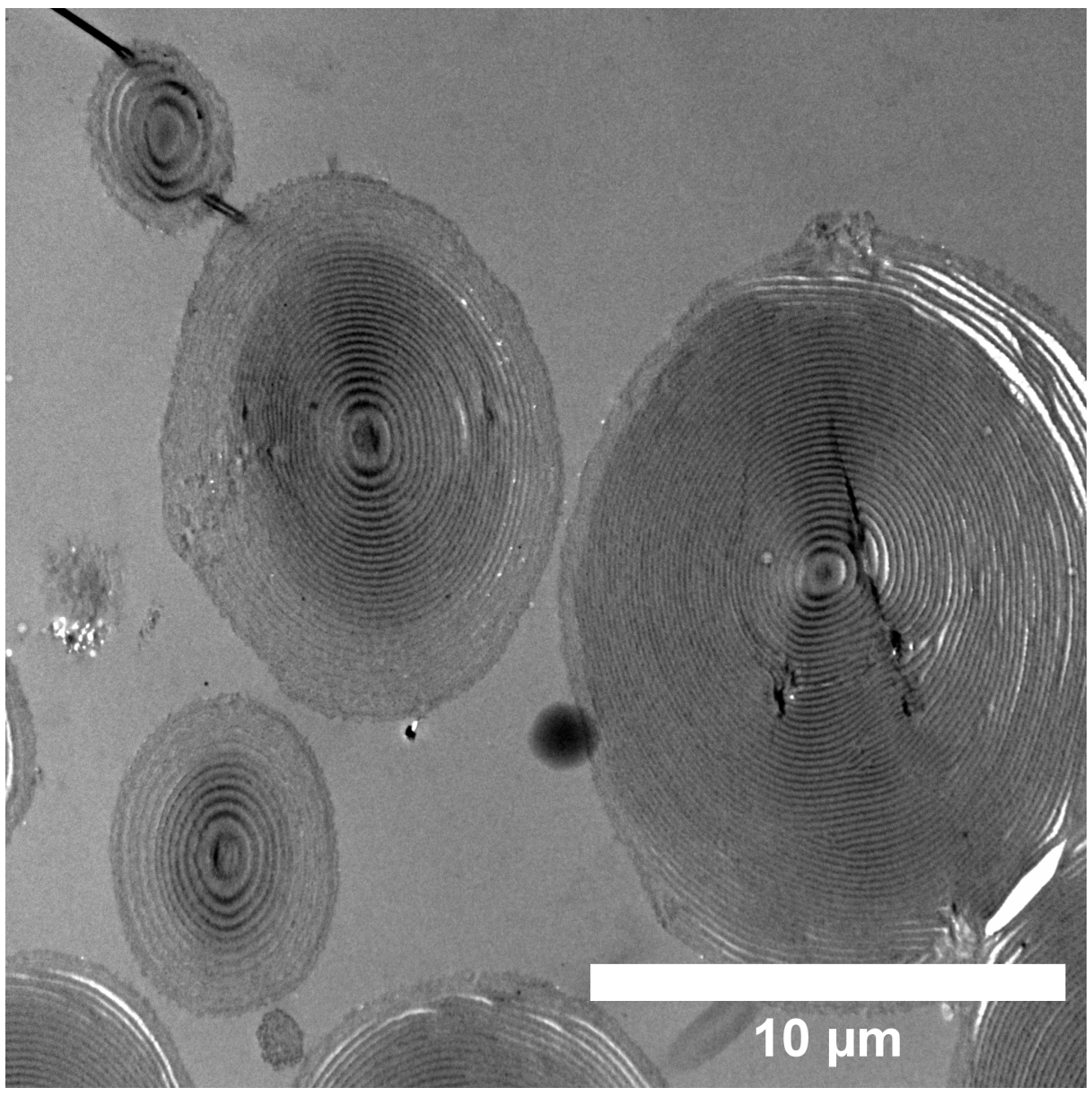

Figure S11: Transmission electron microscopy (TEM) image of microsphere cross-sections containing $f_{NP} = 45\,\%$ TiO_2-HA/DA incorporated in PS_{185kDa}-b-$P2VP_{195kDa}$. The spheres shown are representative of the batch. Microspheres larger than 12 µm in diameter yet exhibit self-assembly into an ordered concentric lamellar phase.

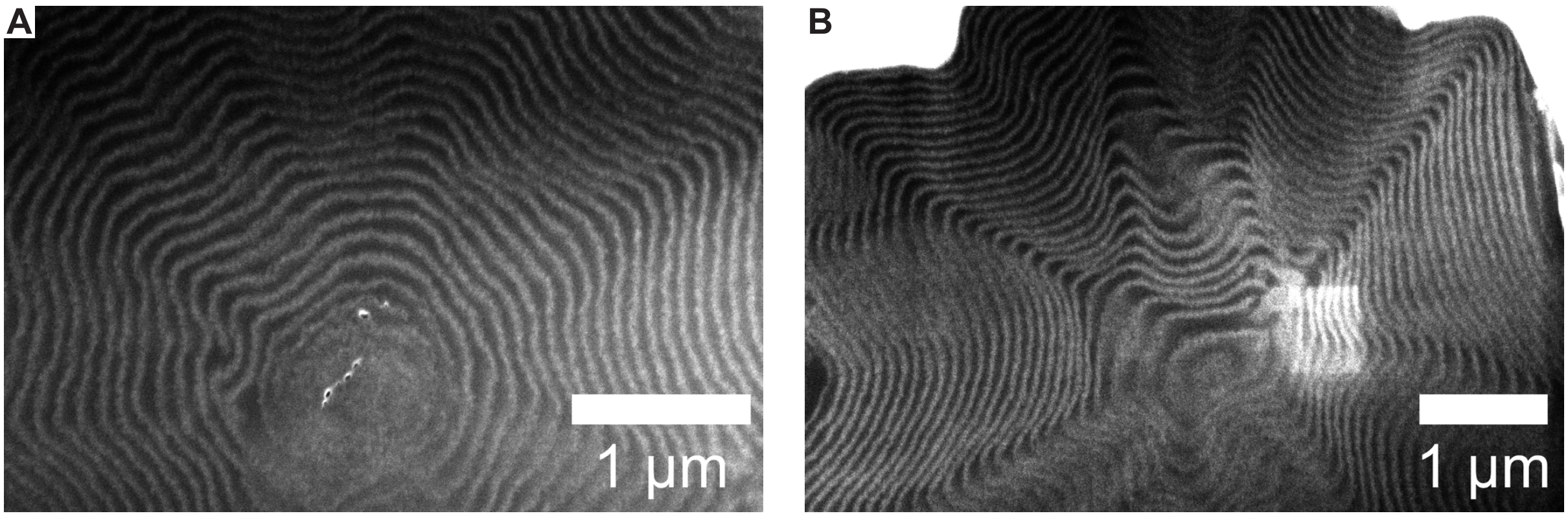

Figure S12: FIB-SEM cross-sectional micrographs of PS_{185kDa}-b-$P2VP_{195kDa}$ microspheres incorporating TiO_2-HA/DA at nanoparticle loadings of $f_{NP} = 90\,\%$ (**A**) and $f_{NP} = 150\,\%$ (**B**). Lamellar wrinkling emerges at $f_{NP} = 90\,\%$ and intensifies with increasing nanoparticle content. No wrinkling is observed at $f_{NP} = 70\,\%$ ($\phi_{NP} = 42\,\%$); see Figure S6.

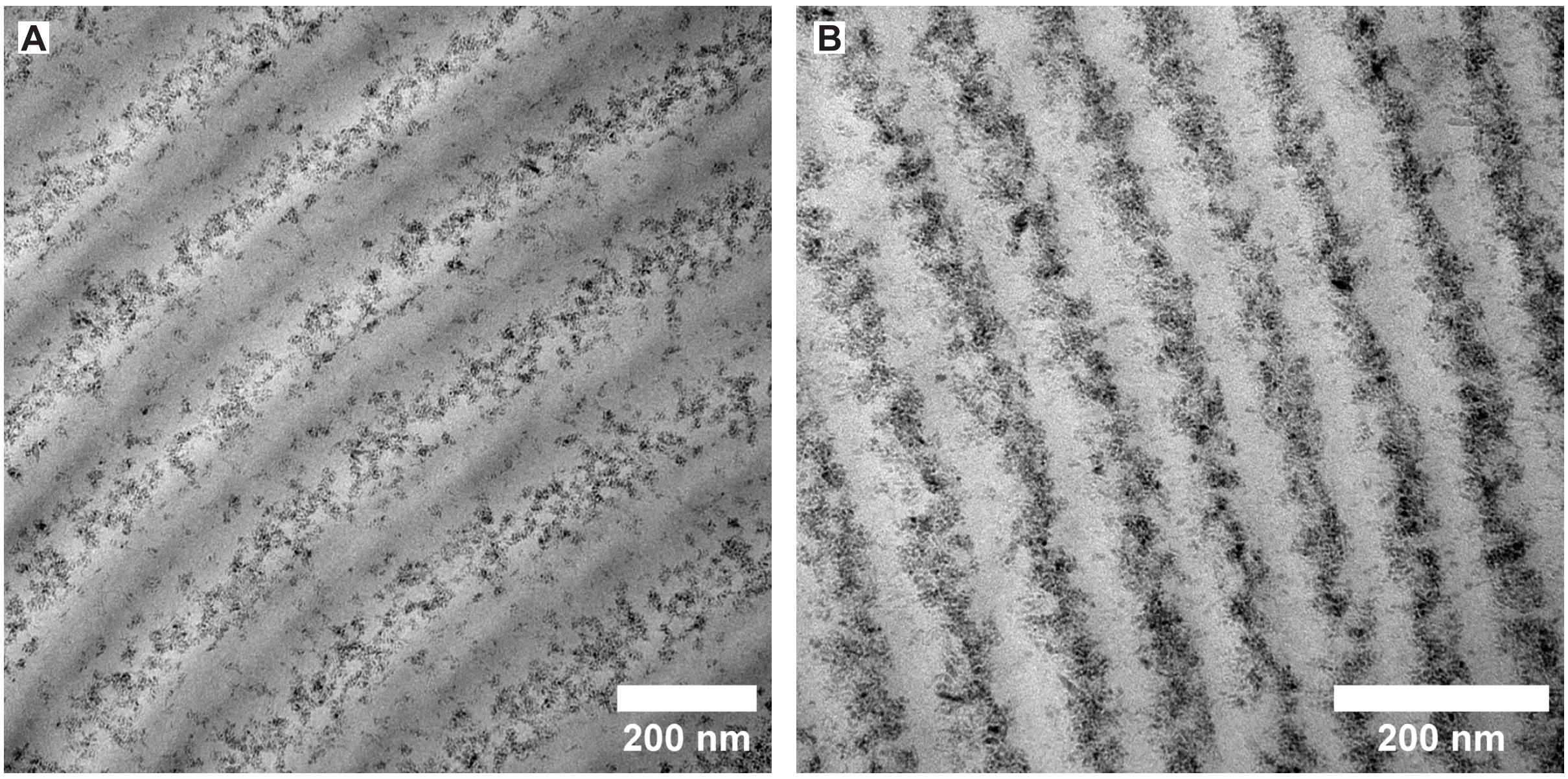

Figure S13: **A** TEM cross-sectional image of PS_{185kDa}-b-$P2VP_{195kDa}$ microspheres loaded with a nanoparticle mass fraction of $f_{NP} = 45\,\%$ TiO_2-HA/DA, showing the lamellar morphology and overall nanoparticle distribution at high loading. **B** TEM cross-sectional image of PS_{185kDa}-b-$P2VP_{195kDa}$ microspheres with the same nanoparticle mass fraction $f_{NP} = 45\,\%$, swollen with hPS_{35kDa} at a swelling ratio of 20 %. The selective incorporation of hPS_{35kDa} into the PS domains modifies the domain contrast and appears to enhance nanoparticle localization relative to the unswollen reference shown in **A**. This indicates that targeted swelling can modulate domain accessibility and improve nanoparticle selectivity within PS-b-P2VP microspheres.

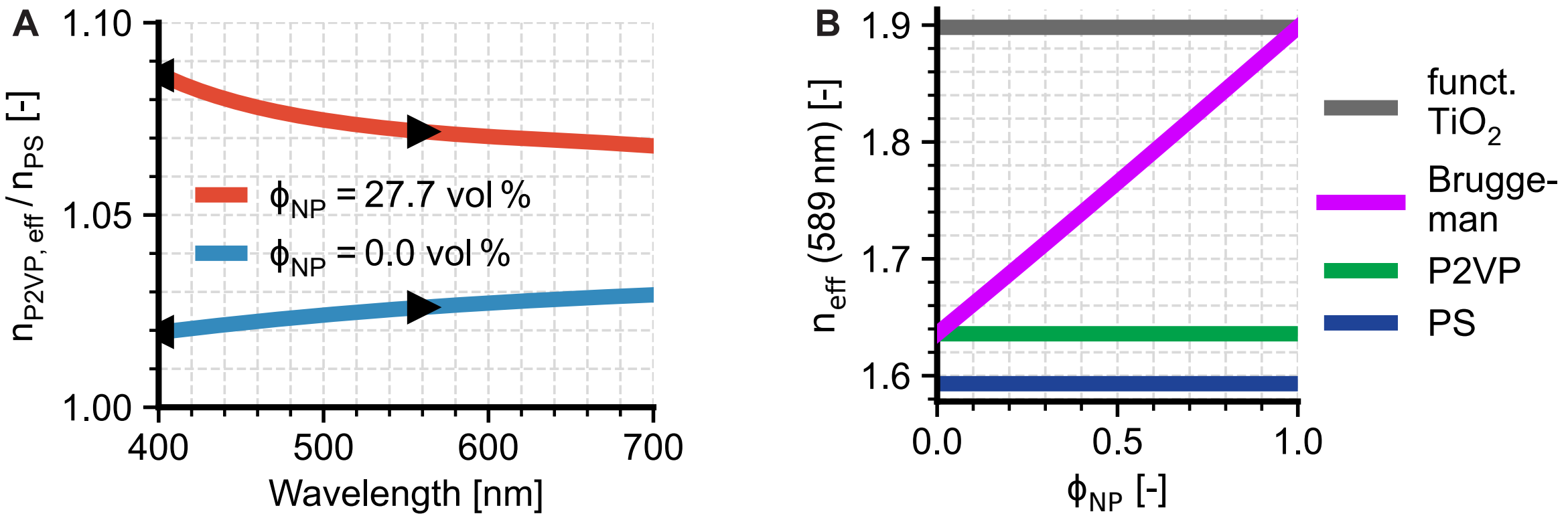

Figure S14: **A** Spectral refractive-index contrast $n_{\mathrm{P2VP,eff}}/n_{\mathrm{PS}}$ for unloaded P2VP and for P2VP containing TiO_2-HA/DA nanoparticles at a volume fraction of $\phi_{\mathrm{NP}} = 27.8\,\%$ (mass fraction $f_{\mathrm{NP}} = 40\,\%$). Triangular markers at 400 nm and 560 nm indicate the reference wavelengths corresponding to Bragg peak positions reported in the optical analysis. **B** Refractive indices of P2VP, PS, and TiO_2-HA/DA at 589 nm. The effective refractive index of the titania–P2VP blend, calculated using the Bruggeman model over the full range of ϕ_{NP}, shows an approximately linear dependence on the nanoparticle volume fraction due to the small intrinsic index contrast between the components.

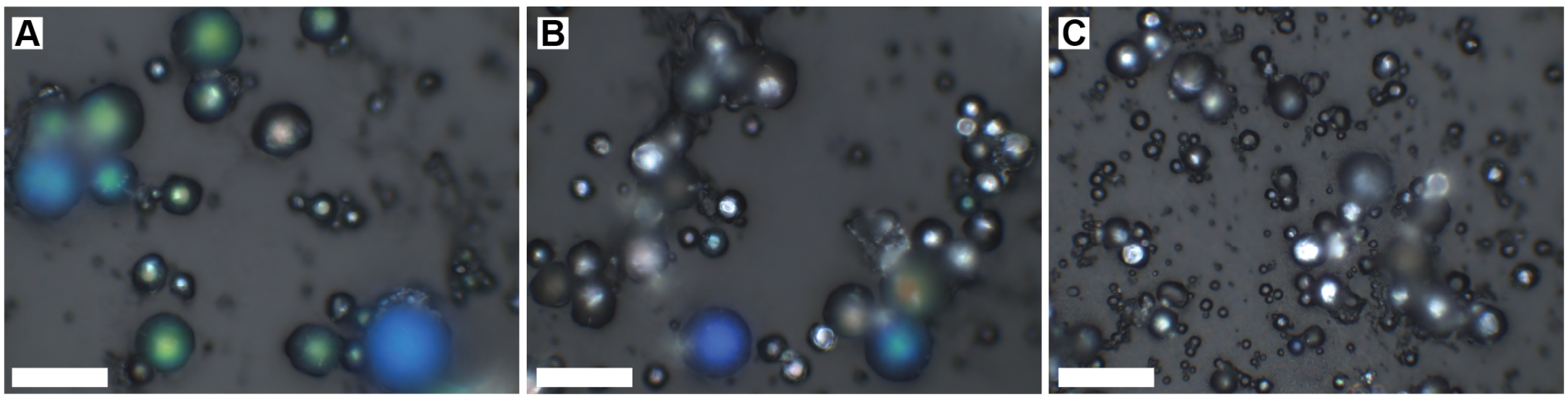

Figure S15: 50× optical micrographs of $\mathrm{PS}_{320\mathrm{kDa}}$-*b*-$\mathrm{P2VP}_{398\mathrm{kDa}}$ microspheres with 40 % TiO_2-HA/DA swollen with $h\mathrm{PS}_{35\mathrm{kDa}}$ at swelling ratios of 25 % (**A**), 50 % (**B**), and 75 % (**C**). Swelling ratios ≥50 % show diminished coloration, suggestive of excess $h\mathrm{PS}_{35\mathrm{kDa}}$ segregation (macrophase separation). Scale bar: 50 µm.

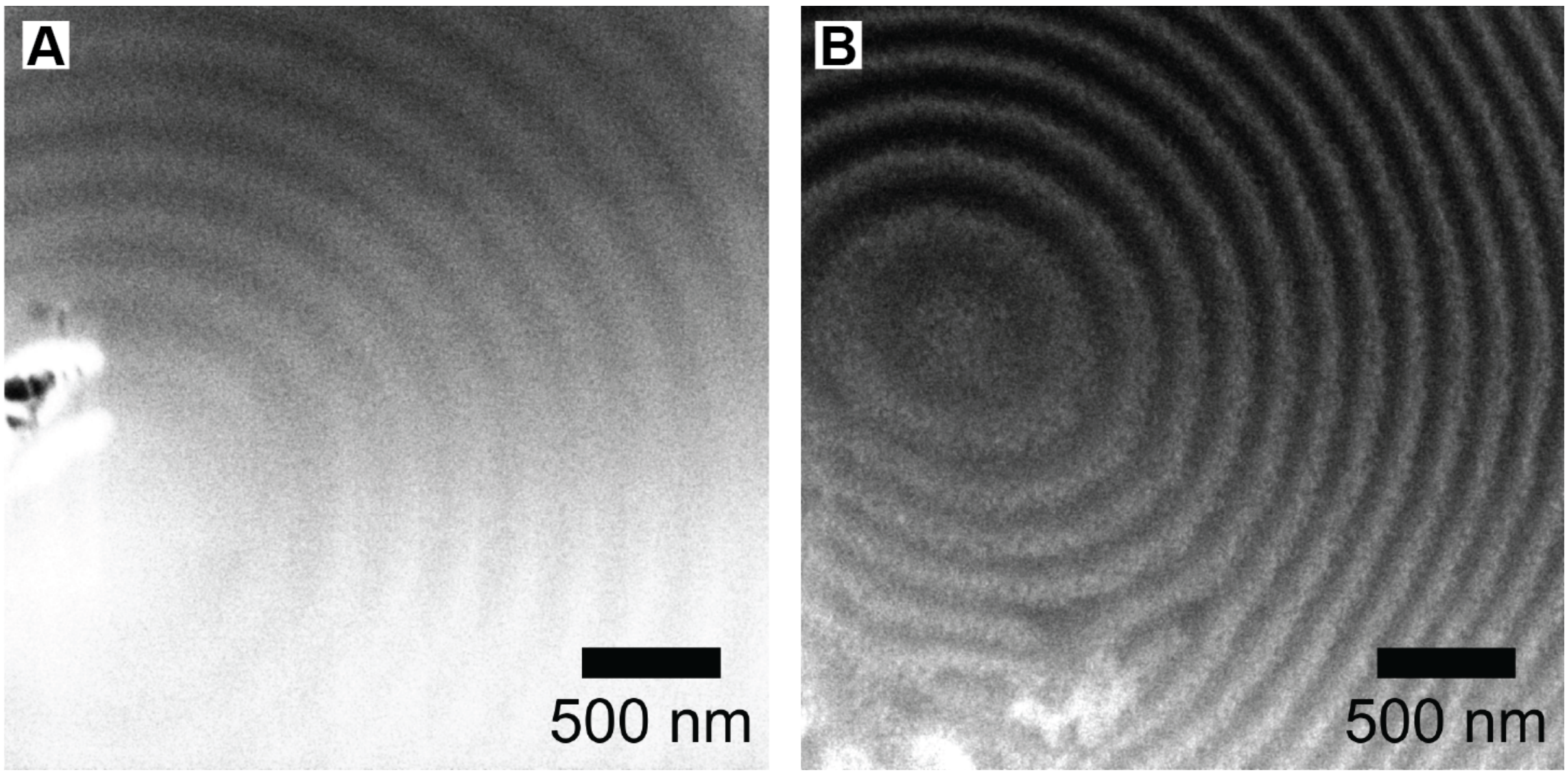

Figure S16: FIB-SEM cross-sectional micrographs of PS_{240kDa}-b-$P2VP_{296kDa}$ microspheres with in **A** none and in **B** $f_{NP} = 40\,\%$ incorporated TiO_2-HA/DA. Concentric lamellae are present and in both cases a swelling ratio of $25\,\%$ $h$$PS_{35kDa}$ is used.

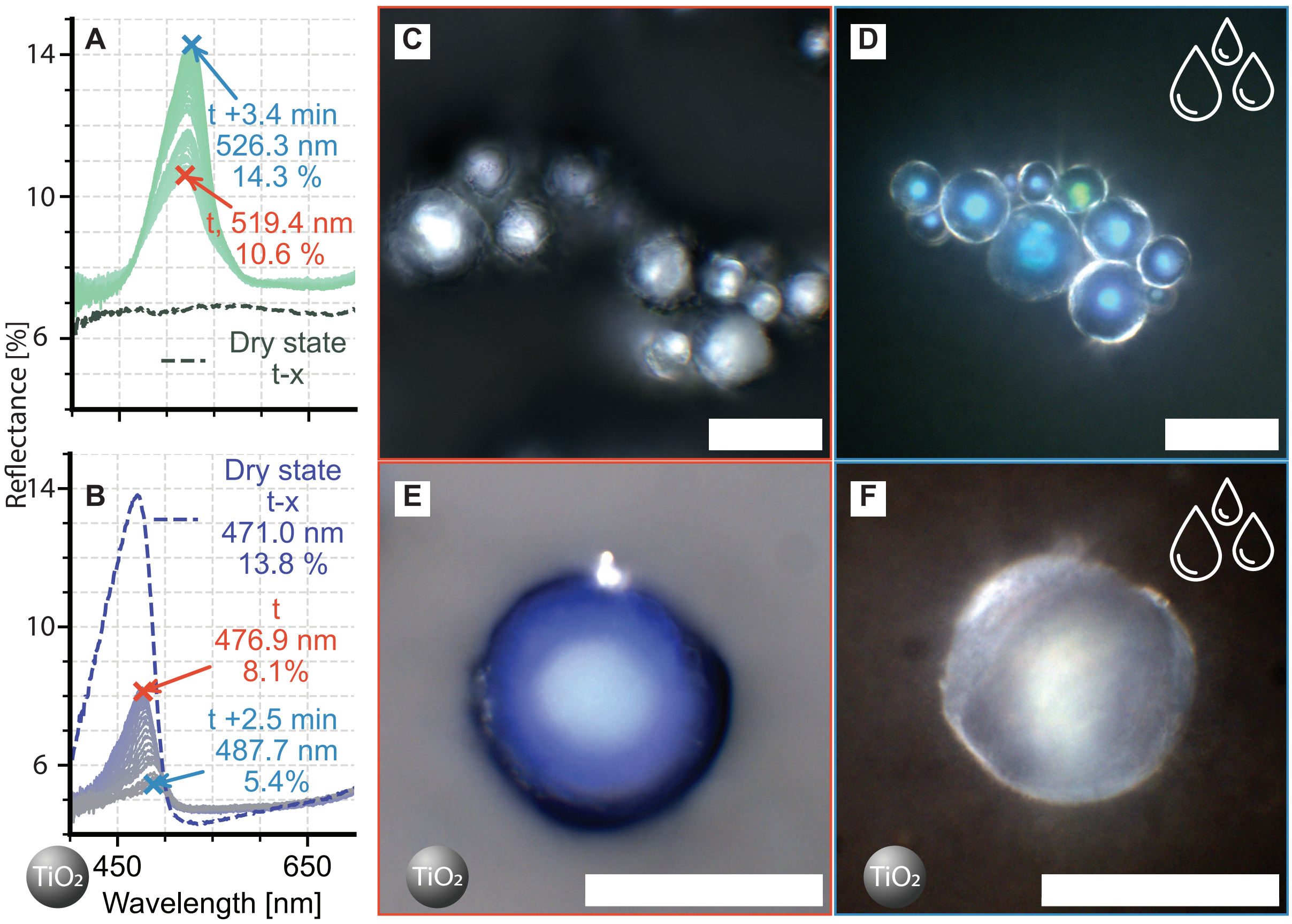

Figure S17: Optical micrographs of photonic pigments in the dry (**C**, **E**) and wet (**D**, **F**) state, prepared *without* (**A**, **C**, **D**) and *with* (**B**, **E**, **F**) TiO_2-HA/DA nanoparticles ($f_{NP} = 25\,\%$ by mass). Time-dependent reflectance spectra for samples without (**A**) and with (**B**) nanoparticles are shown; dashed lines indicate the dry-state spectra. A droplet of water was added after approximately $x \sim 30\,s$, causing a rapid spectral and visual color change that reached equilibrium within $\sim 3\,min$. All optical micrographs were acquired using a 50× objective. Scale bar in **C**–**F**: 50 µm.

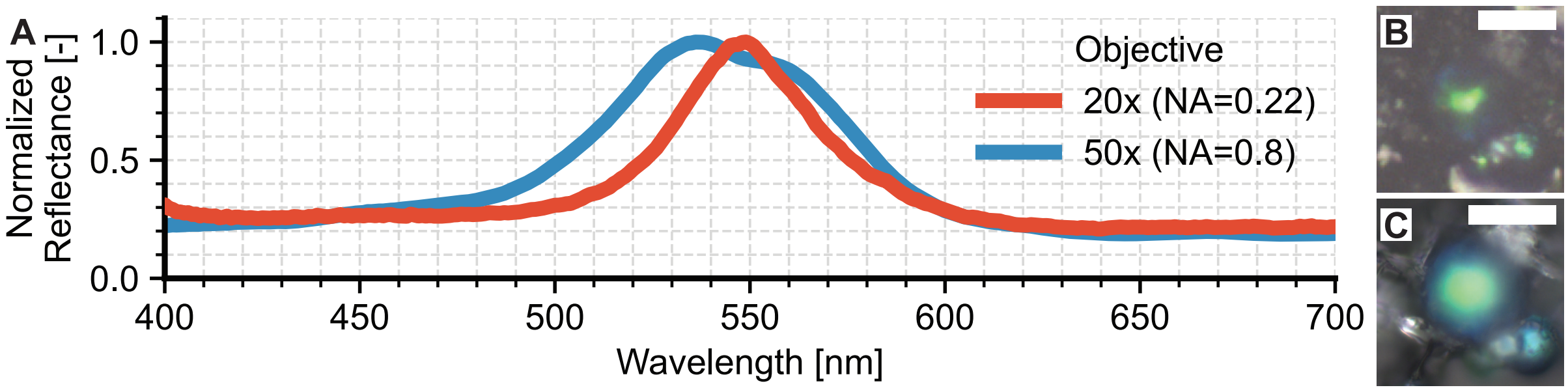

Figure S18: **A** Reflectance spectra recorded from a PS_{185kDa}-*b*-$P2VP_{195kDa}$ microsphere with a swelling ratio of 40 % 2,4,6-triiodophenol. A blue shift is observed for the spectrum acquired at higher numerical aperture (50× objective). **B, C** Optical micrographs of the same microsphere recorded at 20× (**B**) and 50× (**C**). The microsphere diameter is 29.8 µm. Scale bars: 25 µm.

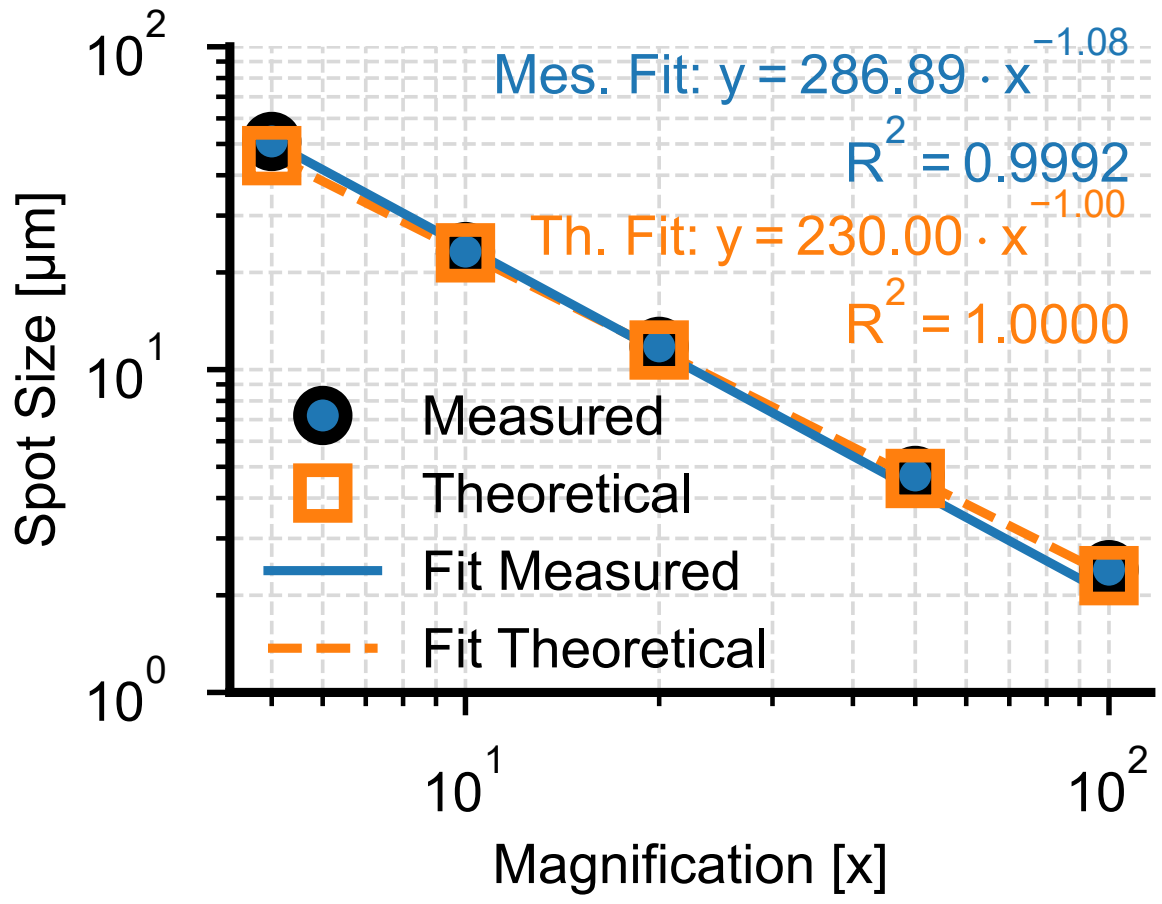

Figure S19: Measured collection area of a 230 µm fiber core as a function of objective numerical aperture (NA). A power-law fit to the measurements is shown and compared with an approximate analytical model.

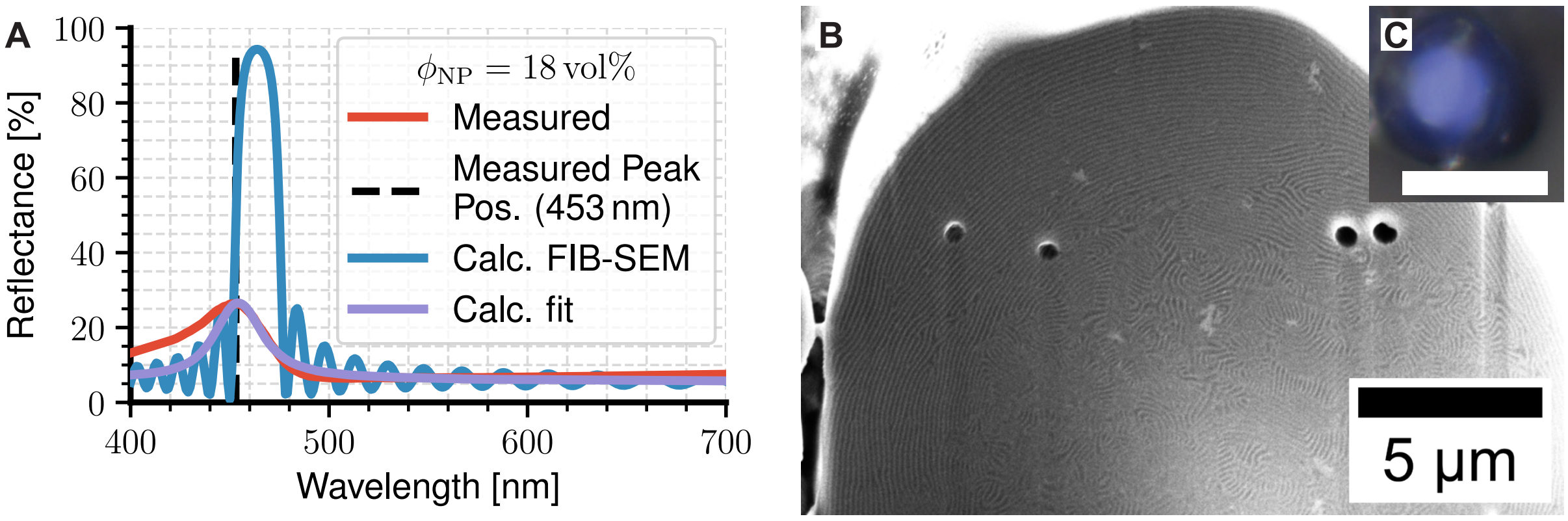

Figure S20: Structure–optics correlation for a single PS_{240kDa}-*b*-$P2VP_{296kDa}$ photonic sphere loaded with TiO_2-HA/DA nanoparticles ($\phi_{NP} = 18.2\,\%$). **A** Measured reflectance spectrum of the selected sphere measured with a 50× objective (NA = 0.8) (red, $\lambda_{max} = 453$ nm, $R_{max} = 26\%$) compared to transfer-matrix calculations for a distributed Bragg mirror (DBR) based on the lamellar stack extracted from the FIB–SEM cross-section (blue; $N_{layers} = 66$, lamellar periodicity $L = 139.5$ nm, $\lambda_{max} = 464$ nm, $R_{max} = 94\%$). The purple curve shows a phenomenological fit using the same number of layers ($N_{layers} = 66$) but allowing the periodicity to vary ($L = 136.3$ nm) and introducing a constant extinction coefficient ($k = 0.045$) to account for optical losses (primarily incoherent scattering). Effective refractive indices were computed via the Bruggeman effective-medium approximation using the wavelength-dependent constituent indices (Figure 4). **B** FIB–SEM cross-section of the same sphere used to determine L and N_{layers} by counting continuous lamellae from the surface up to the first edge dislocation (onset of polycrystalline/disrupted domains). **C** Optical micrograph of the measured sphere (identified via surrounding spheres). Scale bar: 20 µm.

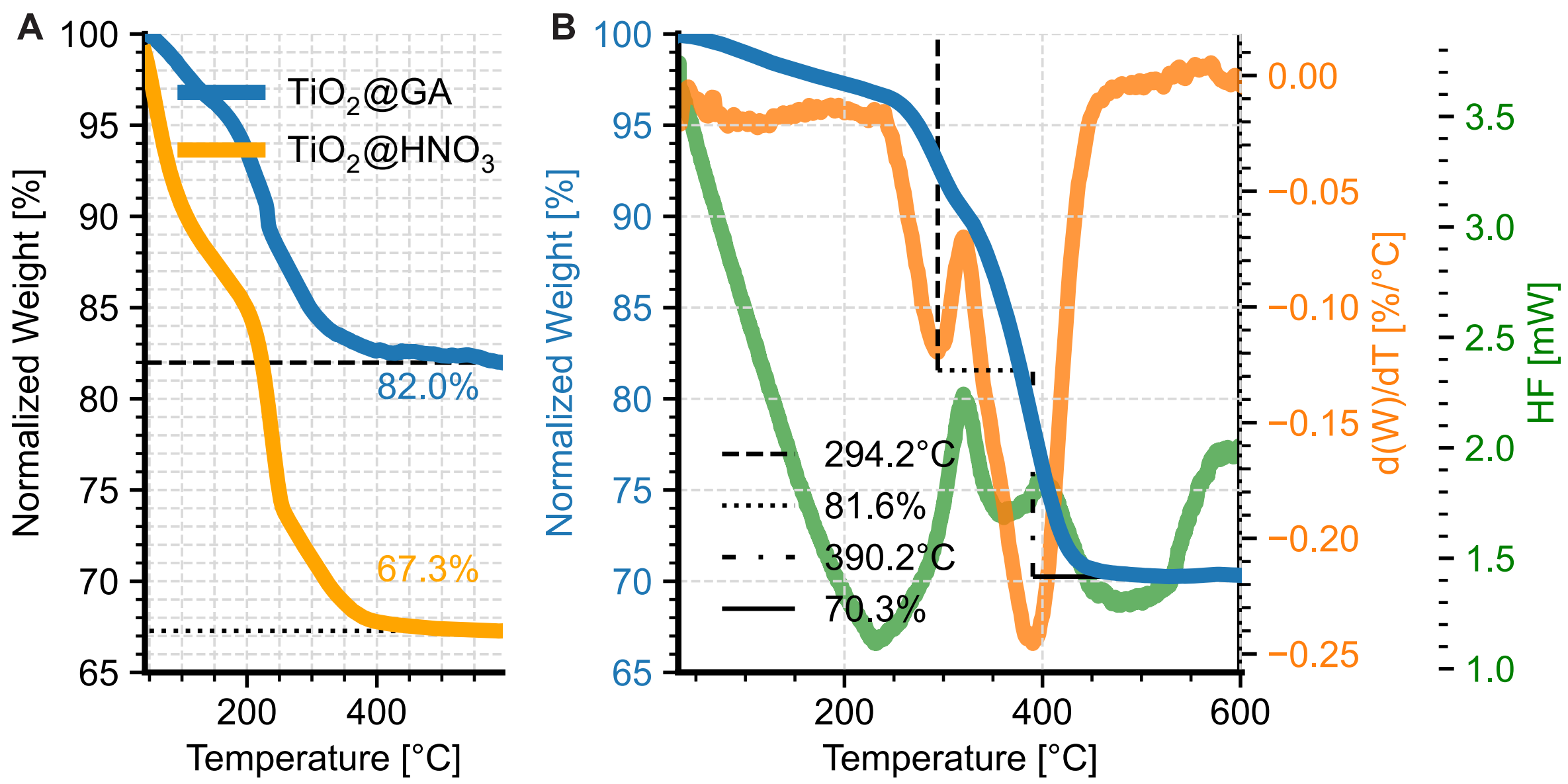

Figure S21: **A** Thermogravimetric analysis of gallic-acid-functionalized titania nanoparticles. Although the initial $TiO_2 - HNO_3$ weight ratio was set to 50:1, the ligand fraction after presumed exchange is 18 %, compared to 32.7 % for the pristine, nitric-acid-covered nanoparticles. **B** Thermogravimetric analysis of commercial TiO_2-HA/DA nanoparticles. The weight curve, its derivative $d(\text{Weight})/d(T)$, and the heat-flow signal are shown, yielding a ligand fraction of 29.7 %.
